# CXCR6+ T cells promote apoptosis and necroptosis in proximal tubules during AKI-to-CKD transition

**DOI:** 10.1038/s41419-026-08644-x

**Published:** 2026-03-24

**Authors:** Xiaoxu Li, Isabel Melchinger, Yuchu Chen, Jiankan Guo, Lloyd G. Cantley, Leyuan Xu

**Affiliations:** Department of Internal Medicine/Section of Nephrology, Yale University School of Medicine, New Haven, CT USA

**Keywords:** Chronic kidney disease, Cell death and immune response

## Abstract

Acute kidney injury (AKI) can progress to chronic kidney disease (CKD) in the setting of maladaptive repair characterized by tubular atrophy, inflammation, and fibrosis. Programmed cell death is a key driver of proximal tubule (PT) loss, yet how immune infiltration promotes tubular injury and death remains incompletely understood. Using a mouse model of maladaptive repair, we integrated bulk and single-cell RNA sequencing with immunohistochemistry and protein analyses to define immune-epithelial interactions during AKI-to-CKD transition. Injured kidneys exhibited loss of healthy PTs, expansion of injured PT subsets, and late-stage T cell accumulation. Apoptotic and necroptotic signaling pathways were markedly upregulated, particularly in VCAM1+ PT cells. Cell-cell interaction analysis identified macrophage-derived *Cxcl16* as the dominant chemokine mediating recruitment of *Cxcr6*+ T cells. Genetic deletion of *Cxcr6* reduced renal T cell accumulation, cytotoxic effector expression, and activation of apoptotic (cleaved caspase-3, Bax) and necroptotic signaling (MLKL, phospho-MLKL) in PT cells. Accordingly, *Cxcr6*^−/−^ mice displayed preserved PT differentiation, reduced fibrosis, and improved renal function. Together, these findings identify *Cxcr6*+ T cells as key mediators of immune-driven tubular cell death during maladaptive repair and suggest that targeting the CXCL16-CXCR6 axis may mitigate tubular injury and slow AKI-to-CKD progression.

## Introduction

Acute kidney injury (AKI) is a common clinical condition characterized by a rapid decline in renal function, often resulting from ischemic, toxic, or inflammatory insults. While the kidney is able to recover from mild AKI, severe or recurrent injury can lead to maladaptive repair, ultimately contributing to the development of chronic kidney disease (CKD) [1]. The transition from AKI to CKD is a complex process involving immune cell infiltration, persistent inflammation, tubular atrophy, and eventual tubulointerstitial fibrosis [2]. A key feature of maladaptive repair is the induction of programmed cell death, including apoptosis [3], necroptosis [4], ferroptosis [5], and pyroptosis [6] in proximal tubule (PT) cells, which exacerbates tissue loss and promotes CKD progression.

T cells have emerged as important mediators of kidney injury [7–9], yet the mechanisms governing their recruitment and cytotoxic activity in the injured kidney remain incompletely understood. Chemokines and their receptors are central regulators of immune cell trafficking, and CXCL16, which binds the receptor CXCR6, is expressed on T cells, including CD8+ T cells [10–12]. CXCR6+ T cells can be recruited to sites of inflammation, where they contribute to tissue damage via cytotoxic and pro-inflammatory pathways, potentially amplifying PT cell death [13].

In this study, we investigated the role of *Cxcl16*-*Cxcr6* signaling in maladaptive kidney repair following AKI, focusing on its contribution to PT cell programmed death. Using single-cell RNA sequencing (scRNA-seq) and in vivo models of ischemia/reperfusion injury (IRI), we characterized interactions between CXCL16-expressing myeloid cells and CXCR6+ T cells, and evaluated how CXCR6 deficiency impacts T cell recruitment, tubular cell death, and renal functional recovery. Our findings provide novel mechanistic insights into how immune-mediated cell death drives AKI-to-CKD progression and highlight potential therapeutic targets to preserve renal function.

## Methods

### Animal surgery and experimental protocol

All animal protocols were approved by the Yale University Institutional Animal Care and Use Committee (IACUC; Protocol ID #: 20493). All methods were performed in accordance with the relevant guidelines and regulations.

C57BL/6J *wild-type* (*WT*) and *Cxcr6*^*-/-*^ male mice (Jackson Laboratory, Strain #:005693) (age 9–10 weeks) were used in this work. All mice were maintained on a 12-h light and 12-h dark cycle with free access to standard rodent chow and water before and after surgery. Due to the substantial gender difference in susceptibility to IRI injury between male and female mice [14], male mice were exclusively used to reduce total numbers of mice required for statistical analysis. Before surgery, all mice were subjected to anesthesia by intraperitoneal injection with ketamine (100 mg/kg) and xylazine (10 mg/kg) as well as phosphate-buffered saline (1× PBS) and subcutaneous injection with Etiqa buprenorphine (3.25 mg/kg) to avoid dehydration and postoperative pain, respectively. To establish the unilateral IRI (U-IRI) model, mouse abdomen was opened, and warm renal ischemia was induced using a nontraumatic microaneurysm clip (FST Micro Clamps) on the left renal pedicle for 17 min on a 37 °C warming pad, leaving the right kidney intact. Blood was collected by retroorbital bleeding on day 1 after U-IRI for serum KIM-1 analysis. Mice were sacrificed 1 day (*n* = 5 mice/genotype) or 14 days (*n* = 8 mice/genotype) after U-IRI, and kidneys were harvested for RNA, protein, and histology analysis. Age-matched uninjured mice were sacrificed as baseline controls (*n* = 8 mice for each genotype). To determine the function of the injured kidney, the mouse dorsal flank was opened, and nephrectomy of the right kidney was performed on day 14 after U-IRI. Blood was collected on days 1, 3, 7, and 14 after contralateral nephrectomy for blood urea nitrogen (BUN) measurement using Stanbio™ BUN liquid reagent for diagnostic set (Thermo Fisher Scientific) and serum creatinine measurement using LC-MS/MS by Yale O’Brien Kidney Center.

### ELISA of serum kidney injury molecule-1 (KIM-1) level

Mouse blood was collected at the indicated time points. Serum KIM-1 concentrations were measured using the mouse TIM-1/KIM-1/HAVCR Quantikine ELISA Kit (R&D Systems) according to the manufacturer’s instructions.

### Histology

Kidneys were fixed in 10% formalin and embedded in paraffin processed for histology. To quantify tubular casts, deparaffinized kidney sections were stained with hematoxylin and eosin (H&E). For detection of collagen, deparaffinized kidney sections (5 µm) were rehydrated, stained with Picrosirius red in 1.3% picric acid for 1 h. Slides were then scanned at the Yale Pathology Tissue Services core facility and processed using ImageScope software. The percent area of cast or Picrosirius red staining was quantified using ImageJ (National Institutes of Health, NIH).

### Immunohistochemistry (IHC) and Immunofluorescence (IF)

Formalin-fixed and paraffin-embedded kidney sections were deparaffinized, rehydrated, antigen retrieved, and stained with primary monoclonal antibodies against CD3ε and CD8α (#99940 and #98941, respectively; Cell Signaling Technology), SOX9 (#82630, Cell Signaling Technology), or biotinylated Lotus Tetragonolobus Lectin (LTL) (#B-1325-2, Vector Laboratories). After washing with TBST, the sections were incubated with biotinylated secondary antibody (Vector Laboratories) followed by VECTASTAIN Elite ABC system (Vector Laboratories). DAB (Vector Laboratories) and hematoxylin (Vector Laboratories) were used as the chromogen and the nuclear counterstain, respectively. IHC staining for TUNEL was conducted by Yale Pathology Tissue Services (YPTS). Whole-slide scanning was conducted by YPTS. LTL-positive area in the cortex was quantified by ImageJ. The number of CD3ε-, CD8α-, TUNEL-, or SOX9-positive cells was counted per high-power field (HPF) from six independent fields per kidney.

Cleaved caspase 3, phospho-MLKL, KIM-1, VCAM-1, LTL, CXCR6, and F4/80 were detected by IF using primary antibodies against, cleaved caspase 3 (clone: 5A1E, Cell Signaling Technology), MLKL (phospho S345) (#37333, Cell Signaling Technology), KIM-1 (#AF1817, Novus Biologicals), VCAM-1 (#32653, Cell Signaling Technology), Ki67 (#12202, Cell Signaling Technology), TUNEL-fluorescein (#11684795910, Roche), LTL-fluorescein (#L32480, Thermo Fisher Scientific), CXCR6 (#NLS1102, Novus Biologicals), and F4/80 (#MCA497, Bio-Rad), respectively. The sections were counterstained with DAPI (Sigma-Aldrich) and mounted with VECTASHIELD® HardSet™ Antifade Mounting Medium. The fluorescence images were obtained by confocal microscopy (Zeiss LSM 880) using tile-scanning. The percentage of KIM-1- and VCAM-1-positive area was quantified using ImageJ.

### In vitro cell culture

#### Isolation and culture of bone marrow-derived macrophages (BMMs)

BMMs were isolated from *Wild-type* or myeloid differentiation factor 88/TIR-domain-containing adapter-inducing interferon-β (*Myd88*^*−/−*^*;Trif*^*−/−*^) mice using our recently reported protocol [15, 16]. Briefly, bone marrow cells were flushed from the femurs of 8–10-week-old mice, and red blood cells were lysed in RBC lysis buffer (Thomas Scientific). Remaining cells were incubated in RPMI 1640 medium (Life Technologies) supplemented with 10% FBS (Life Technologies), 10 μM macrophage-colony stimulating factor (M-CSF) (GenScript), 1% glutamine (Life Technologies), 1% MEM vitamin (Life Technologies), and 1% penicillin/streptomycin (Life Technologies) in regular tissue culture dishes. After 24 h, nonadherent cells (containing the majority of BMMs) were transferred to a new petri dish and incubated for an additional 7 days (with one medium change on day 3) to generate naïve BMMs.

Cultured BMMs were detached with 2 mM EDTA in PBS and plated in 6-well plates in the above mentioned complete media for 24 h. *Wilt-type* BMMs were treated with one of the following agent: 500 µM H_2_O_2_, 25 ng/mL GM-CSF (R&D Systems), 100 ng/mL interferon γ (R&D Systems), 20 ng/mL TNFα (R&D Systems), or 20 ng/mL IL-1β (R&D Systems) for 6 h. In a separated experiment, *wild-type* BMMs were treated with TNFα (20 ng/mL) or IL-1β (20 ng/mL) in the presence or absence of a selective IKKα and IKKβ inhibitor, ACHP (1 µM) (R&D Systems) for 6 h. *Myd88*^*−/−*^*;Trif*^*−/−*^ BMMs were treated with TNFα (20 ng/mL) or IL-1β (20 ng/mL) for 6 h. After treatment, cell lysates were prepared with RLT buffer (Qiagen) supplemented with fresh β-mercaptoethanol for RNA extraction.

#### Culture of mouse proximal tubule cells (MPTs) and primary cultured renal cells (PCRCs)

Pathogen-free immortalized mouse proximal tubule (MPT) cells were plated in 6-well plates in Dulbecco’s modified Eagle medium (DMEM) (Gibco) supplemented with 10% FBS and antibiotic-antimycotic (Gibco) for 24 h. Cells were then treated for 6 h with one of the following: MPT cell debris, 500 µM H₂O₂, TNF-α (20 ng/mL), IFN-γ (100 ng/mL). Following treatment, cells were lysed in RLT buffer supplemented with freshly added β-mercaptoethanol for RNA extraction.

PCRCs were isolated from *Wild-type* or *Myd88*^*−/−*^;*Trif*^*−/−*^ mice using our recently reported protocol [17]. Briefly, kidneys were harvested and minced into 2 mm^3^ cubes with a razor blade, and digested in Liberase (5 mL per kidney, 0.5 mg/mL; Roche), supplemented with DNase I (100 µg/mL, Roche) and MgCl_2_ (0.1%) in PBS for 30 min, with gentle pipetting every 10 min. The digested tissue was passed through a 70 µm cell strainer (Falcon) into a 50 mL conical tube, rinsed with 45 mL ice-cold PBS, and centrifuged at 1000 rpm for 5 min at 4 °C. The resulting pellet, containing single cells and tubular fragments, was resuspended with 3 mL red blood cell lysis buffer (Alfa Aesar) and incubated at room temperature for 3 min to remove residual erythrocytes. Cells were then cultured in complete medium consisting of DMEM supplemented with 10% FBS and Antibiotic-Antimycotic solution. After an initial medium change 48 h after seeding, cultures were maintained for an additional 3 days. PCRCs were subsequently trypsinized and replated in 6-well plates and allowed to adhere for 24 h.

In a separate experiment, MPT cells or PCRCs were treated with TNF-α (20 ng/mL) or IL-1β (20 ng/mL) in the presence or absence of the selective IKKα/IKKβ inhibitor ACHP (1 µM) for 6 h. Following treatment, cells were lysed in RLT buffer supplemented with freshly added β-mercaptoethanol for RNA extraction.

#### Quantitative PCR analysis

RNA from whole kidney or cell lysate was extracted with RNeasy Mini kit (Qiagen) and reverse transcribed using the iScript cDNA synthesis kit (Bio-Rad). Gene expression levels were determined by quantitative real-time PCR using the iCycler iQ (Bio-Rad) and normalized to *Hprt1*. Primer sequences are provided in Supplementary Table 1. Data were expressed using the comparative threshold cycle (ΔCT) method, and the relative mRNA levels were presented by 2^−ΔCT^ or ΔΔCT.

#### Western blot analysis

Kidney lysates from *wild-type* and *Cxcr6*^*−/−*^ mice were prepared using radioimmunoprecipitation assay (RIPA) lysis and extraction buffer (Thermo Fisher Scientific) supplemented with 1× protease and phosphatase inhibitor cocktail (Roche). Protein concentrations were determined using the Bio-Rad Protein Assay. Equal amounts of protein (50 μg per sample) were separated by SDS–PAGE on 10% or 12% polyacrylamide gels (Bio-Rad) and transferred to Immobilon PVDF membranes (Millipore). Membranes were blocked with 5% nonfat milk or 5% bovine serum albumin (BSA) in Tris-buffered saline with 0.1% Tween-20 (TBST) for 2 h at room temperature and incubated overnight at 4 °C with primary antibody against cleaved caspase-3 [clone 5A1E (#9664) or #9661, Cell Signaling Technology]. After TBST washes, membranes were incubated for 1 h at room temperature with horseradish peroxidase (HRP)-conjugated secondary antibody (Thermo Fisher Scientific). Protein bands were visualized using the ECL detection system (Thermo Fisher Scientific) and imaged with the LI-COR Odyssey FC Imager. Membranes were subsequently washed with TBS, stripped in Restore PLUS Western Blot Stripping Buffer (Thermo Fisher Scientific) for 15 min, rewashed with TBST, and re-blocked. The same membranes were sequentially re-probed with primary antibodies against Bax (clone D3R2M, Cell Signaling Technology), MLKL (clone E7V4W, Cell Signaling Technology), phospho-MLKL (Ser345, clone EPR9515(2), Abcam), RIP3 (clone D4G2A, Cell Signaling Technology), and HSP90 (clone C45G5, Cell Signaling Technology), following the same protocol. Band intensity was quantified using ImageJ.

#### Bulk RNA sequencing (RNA-seq) data analysis of mouse IRI kidneys

Total RNA was extracted from control kidneys (*n* = 4) and injured kidneys (*n* = 5) collected on day 14 post-IRI using the RNeasy Mini kit (Qiagen) supplied with on-column RNase-free DNase I treatment (Qiagen). Library preparation was performed using Illumina’s standard PolyA enrichment protocol by the Yale Center for Genome Analysis. Sequencing was carried out on the NovaSeq platform with 100-bp paired-end reads at a depth of 50 million reads per sample. Raw reads were aligned to the mouse genome (mm10) with STAR [18], and gene expression levels were quantified with FeatureCounts [19]. Differential expression analysis between the different groups was conducted with DESeq2 [20]. Genes with an adjusted *P* < 0.05 were considered significantly differentially expressed. DEGs with a log2 fold change >0.25 were subjected to gene set enrichment analyses using ClusterProfiler and KEGG R packages [21].

#### Single cell-RNA sequencing (scRNA-seq) data analysis of mouse IRI kidneys

We re-analyzed our previously published scRNA-seq dataset (GSE197626) from the U-IRI mouse kidney model (on days 14 and 30) and control kidney using the Seurat v5.1.0 R package [22, 23]. Cells of poor quality were excluded based on the following criteria: <500 unique genes or <1000 counts (likely cell fragment), or >7500 unique genes (potentially cell duplet). Additionally, cells with mitochondrial gene content exceeding 50% and low-complexity cells, such as red blood cells with <0.8 log10 genes per UMI counts, were removed. Only genes expressed in at least five cells were retained for further analysis. Doublets were identified and excluded using the DoubletFinder R package [24]. Confounding factors, including mitochondrial genes, were removed before downstream clustering [25]. After applying quality control filters, a total of 30,323 cells remained, with a median of 5301 counts per cell and a sequencing depth of 23,438 genes across all cells. The dataset was normalized, scaled, and integrated using reciprocal principal component analysis (RPCA) on the 2000 most variable genes. The top 30 principal components were chosen for cell clustering and neighbors finding with k.param = 20. Dimensionality reduction was performed by retaining the top 30 principal components, and cell clustering was carried out with k.param = 20. Uniform manifold approximation and projection (UMAP) was used for two-dimensional visualization. Marker genes for each cluster were identified through differential expression analysis using the Wilcoxon rank-sum test, considering genes expressed in at least 25% of cells either within or outside the cluster. This analysis identified 30 distinct cell clusters based on kidney and immune cell lineage-specific marker expression. Cell interaction analysis was performed using CellChat v2 R package [26]. Finally, the relative expression levels of *Ccl6*, *Ccr2*, *Cxcl16*, and *Cxcr6* were analyzed across all the cell clusters and time points. Gene set enrichment analysis across multiple PT clusters was analyzed using the singleseqgset R package.

### Statistical analysis

All in vitro experiments were repeated at least three times. Data were expressed as means ± standard deviation (SD). Two-group comparison was performed by two-tailed unpaired Student’s *t* test. Multigroup comparison was performed by one-way analysis of variance (ANOVA) for group mean comparison followed by Tukey’s multiple comparison test for subgroup comparison. Correlation of gene expression was performed by Pearson correlation coefficient R with a two-tailed *P* value. All statistical analyses were performed using Prism 10 (GraphPad Software). A value of *P* < 0.05 was considered statistically significant.

## Results

### Upregulation of apoptosis and necroptosis during AKI-to-CKD transition

**Fig. 1:**
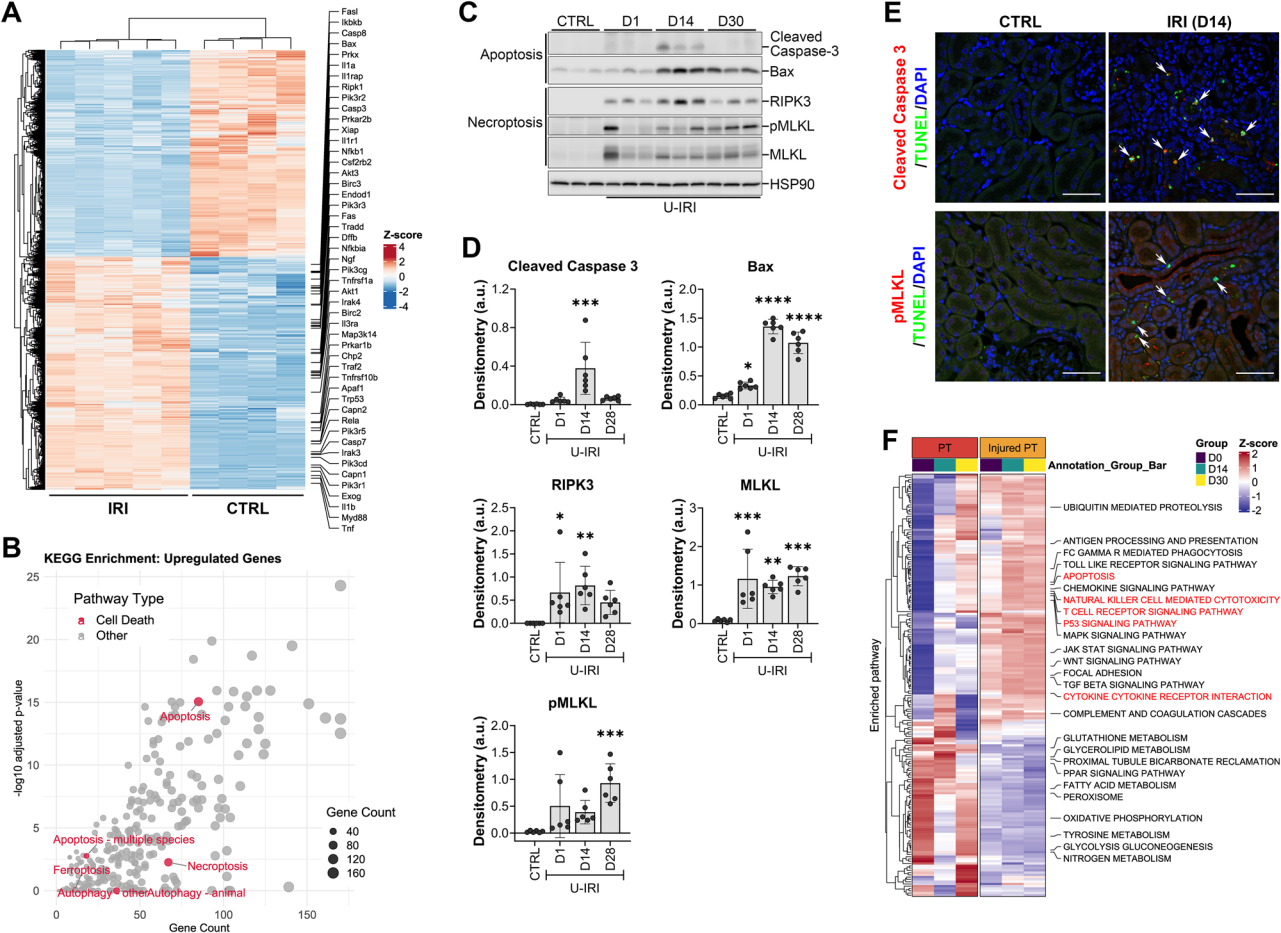
Upregulation of apoptosis and necroptosis signaling pathways during AKI-to-CKD transition. **A**, **B** *Wild-type* mice were subjected to unilateral ischemia/reperfusion injury (IRI), and the injured kidneys were collected on day 14 post-injury for bulk RNA-seq analysis. Age-matched healthy kidneys served as controls (CTRL). **A** Differentially expressed genes (DEGs) were identified using DEseq2 and visualized in a heatmap (*n* = 4 CTRL and *n* = 5 IRI kidneys). **B** KEGG pathway enrichment analysis of upregulated DEGs in IRI kidneys. Cell death pathways (apoptosis, necroptosis, ferroptosis, pyroptosis, and autophagy) are highlighted in the bubble plot. **C**, **D** *Wild-type* mice were subjected to unilateral IRI, and the injured kidneys were harvested on days 1, 14, and 28 post-injury. Age-matched healthy kidneys served as controls (CTRL). **C** Western blot analysis of cleaved caspase 3, Bax, phospho-MLKL, MLKL, RIP3, and HSP90 (loading control, re-probed after stripping) from whole kidney lysates (each lane represents an individual kidney). The full length uncropped original Western blots were provided in Supplementary Fig. 1. **D** Densitometric quantification of protein bands. Expression of cleaved caspase-3, Bax, phospho-MLKL, MLKL, and RIP3 was normalized to HSP90. *n* = 6 kidneys/group. One-way ANOVA: *P* = 0.0004 (cleaved caspase 3), *P* < 0.0001 (Bax), *P* = 0.0034 (phospho-MLKL), *P* = 0.0003 (MLKL), and *P* = 0.0138 (RIP3). *P < 0.05, **P < 0.01, ***P < 0.001, and ****P < 0.0001 by Tukey’s multiple comparison as compared to CTRL. **E** *Wild-type* mice were subjected to U-IRI, and the injured kidneys were harvested on day 14 post-injury. Control (CTRL) kidneys were obtained from healthy uninjured mice. Kidney sections were immunofluorescence-stained for cleaved caspase 3 (upper panel, red), phosphorylated MLKL (pMLKL; lower panel, red), TUNEL (green), and DAPI (blue). Individual channels are shown in Supplemental Fig. 2. Original magnification, ×63. Scale bar: 50 μm. (**F**) *Wild-type* mice were subjected to unilateral (IRI), and the injured kidneys were collected on days 14 and 30 post-injury for single-cell RNA sequencing analysis as shown in Supplementary Fig. 2A and 1B. KEGG pathway enrichment analysis was performed on healthy and injured proximal tubule (PT) cells and visualized as a heatmap.

The transition from AKI to CKD is commonly associated with tubular cell death and progressive kidney atrophy [22]. To identify the predominant cell death mechanisms involved, we first performed bulk RNA-seq analysis of kidneys harvested 14 days after IRI. Multiple genes associated with cell death pathways were upregulated in IRI kidneys compared with controls (Fig. 1A). Pathway enrichment analysis revealed apoptosis and necroptosis as the top-enriched cell death pathways (Fig. 1B). To validate these findings, we assessed protein expression of key regulators of apoptosis and necroptosis at the whole-kidney level. Cleaved caspase-3 and Bax showed minimal or modest induction on day 1 but were markedly increased on day 14 post-IRI. In contrast, RIPK3, MLKL, and phosphorylated MLKL were strongly upregulated as early as day 1 and remained highly expressed on days 14 and 28 (Fig. 1C, D). These results suggest that necroptosis is engaged throughout initial injury and maladaptive repair, whereas apoptosis is selectively upregulated at the time of kidney atrophy during the AKI-to-CKD transition. IF co-staining with TUNEL reveals that the majority of TUNEL-positive tubular cells co-express either cleaved caspase-3 or phosphorylated MLKL, confirming the presence of both apoptotic and necroptotic cell death following U-IRI (Fig. 1E and Supplementary Fig. 2). To assess whether cell death pathways were specifically upregulated in PT cells, we re-analyzed our scRNA-seq dataset from mouse kidneys subjected to U-IRI at days 14 and 28 [22]. Consistent with previous reports, we observed a decline in healthy PT populations accompanied by an expansion of injured PT and T cell populations (Supplementary Fig. 3A, B). At the single-cell level, KEGG pathway enrichment analysis confirmed a significant upregulation of apoptosis in injured PT cells, along with activation of natural killer (NK) cell–mediated cytotoxicity, T cell receptor signaling, and cytokine-receptor interactions (Fig. 1F). These findings indicate that PT cells, particularly VCAM1+ subsets, are subject to both apoptosis and immune cell–mediated necroptosis during the AKI-to-CKD transition.

### Identification of T cell homing signals during AKI-to-CKD transition

**Fig. 2:**
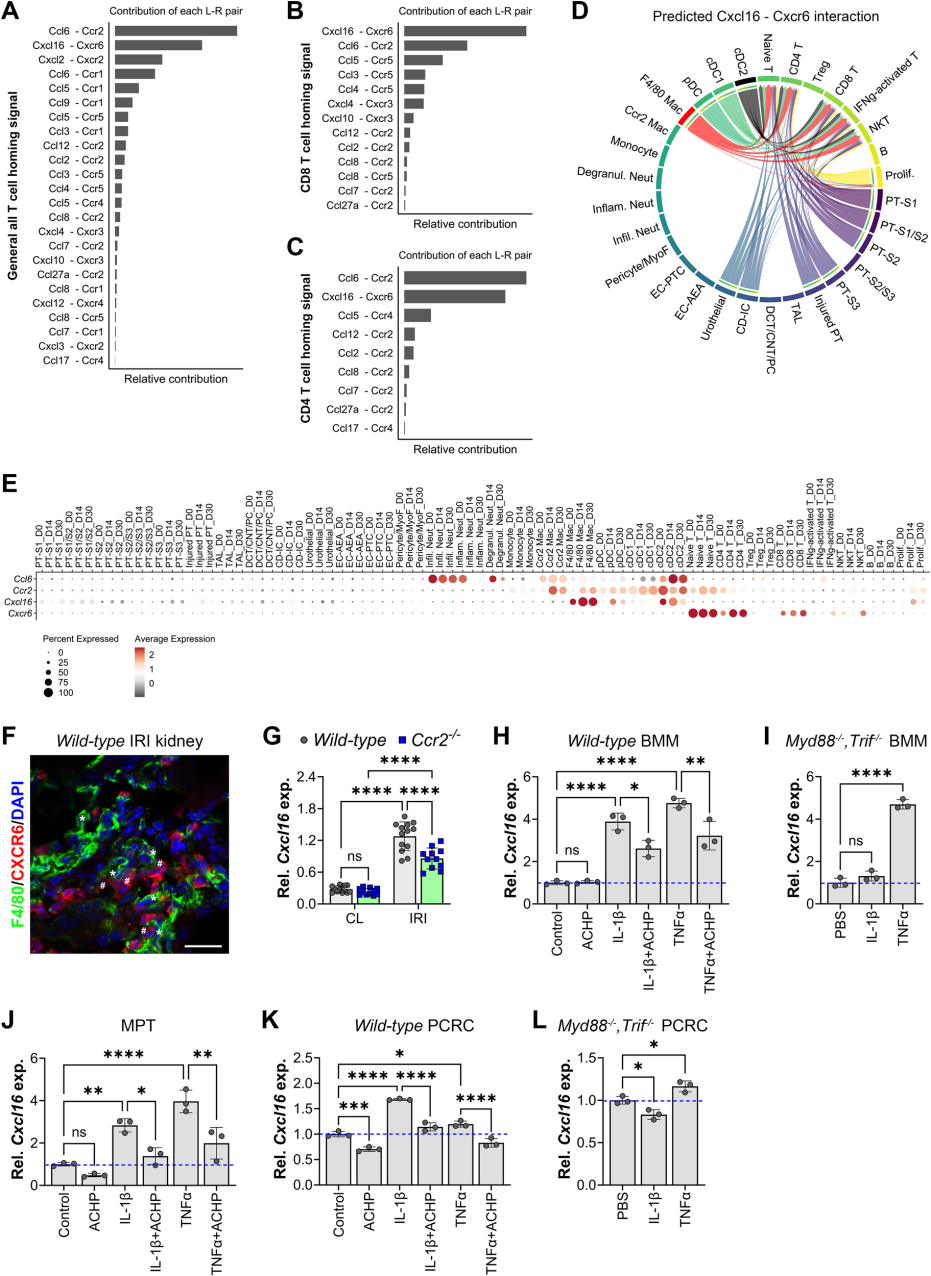
Identification of T cell homing signals during AKI-to-CKD transition. *Wild-type* mice were subjected to unilateral ischemia/reperfusion injury (U-IRI), and the injured kidneys were collected on day 14 and 30 post-injury for single-cell RNA sequencing analysis as shown in Supplementary Figs. 2A and 1B. **A**–**C** Ligand-receptor interaction analysis using CellChat R package. The contribution of each ligand-receptor pair to the overall signaling pathway (CCL and CXCL) in overall T cells (**A**), CD8+ T cell (**B**), and CD4+ T cell (**C**) was computed and visualized in a bar graph, respectively. **D** The cell-cell communication mediated by *Cxcl16*-*Cxcr6* pair was visualized in a chord hierarchy plot. **E** The distribution and relative expression of top pairs of ligand-receptor genes were visualized using a dot plot. PT proximal tubule, TAL thick ascending limb, DCT distal convoluted tubule, CNT connecting tubule, PC principal cell, CD-IC collecting duct-intercalated cell, EC-AEA endothelia cell-afferent/efferent arteriole, EC-PTC peritubular endothelia cell, MyoF myofibroblast, Infil. Neut infiltrating neutrophil, Inflam. Neut inflammed neutrophil, Degranul. Neut degranulated neutrophil, Infil. Mac infiltrating macrophage, Resid. Mac resident macrophage, pDC plasmacytoid dendritic cell, cDC conventional dendritic cell, T T cells, NK natural killer cells, B B cells, Prolif proliferating cells. **F** Kidney cryosections were immunofluorescence-stained with CXCR6 (red), F4/80 (identifies macrophages, green), and DAPI (identifies nuclei, blue) at day 14 after unilateral IRI. Original magnification, ×400. Scale bar: 20 μm. Asterisks (*) indicate representative macrophages adjacent to CXCR6⁺ cells (denoted by #). **G** *Wild-type* and *Ccr2*^*−/−*^ mice were subjected to unilateral IRI. IRI and contralateral (CL) kidneys were harvested 30 days after IRI. Quantitative PCR for *Cxcl16* was performed on whole-kidney mRNA. Two-way ANOVA: *P* < 0.0001 (injury and genotype factors) and *P* = 0.0007 (interaction). ****P < 0.0001 by Tukey’s multiple comparison. ns not statistically significant. *Wild-type* bone marrow-derived macrophages (BMMs, **H**), MPT cells (**J**), and primary cultured renal cells (PCRCs, **K**) were treated with PBS (control), interleukin (IL-1β, 10 ng/mL), or tumor necrosis factor (TNF-α) (20 ng/mL) with or without the NF-κB inhibitor ACHP (1 μM) for 6 h. Quantitative PCR for *Cxcl16* was performed on BMM mRNA. n=3 independently treated experiments. One-way ANOVA: *P* < 0.0001. *P < 0.05, **P < 0.01, ***P < 0.001, and ****P < 0.0001 by Tukey’s multiple comparison. *Myd88*^−^^*/−*^;*Trif*^*−/−*^ BMMs (**I**) and PCRCs (**L**) were treated with PBS (control), IL-1β (20 ng/mL), or TNF-α (20 ng/mL) for 6 h. Quantitative PCR for *Cxcl16* was performed on BMM mRNA. *n* = 3 independently treated experiments. One-way ANOVA: *P* < 0.0001 (BMM) and *P* = 0.0011 (PCRC). *P < 0.05 and ****P < 0.0001 by Tukey’s multiple comparison. ns, not statistically significant.

Previous studies have shown that maladaptive repair is characterized by late T cell infiltration which strongly correlates with kidney atrophy [22, 27]. To identify the signals mediating T cell recruitment, we performed cell-cell interaction analysis using the CellChat R package, focusing on chemokine-chemokine receptor interactions in injured kidneys. This analysis identified *Ccl6*-*Ccr2* and *Cxcl16*-*Cxcr6* as the top ligand-receptor pairs mediating interactions between T cells and other cell types (Fig. 2A). Specifically, *Cxcl16*-*Cxcr6* was the predominant homing signal for CD8+ T cells (Fig. 2B), whereas *Ccl6*-*Ccr2* primarily mediated recruitment of CD4+ and naïve T cells (Fig. 2C and Supplementary Fig. 3C). Further analysis revealed that F4/80+ macrophages were the main source of *Cxcl16* driving CXCR6+ CD8+ T cell recruitment during the AKI-CKD transition (Fig. 2D); whereas myeloid cells broadly supported CD4+ T cell recruitment via *Ccl6*-*Ccr2* signaling (Supplementary Fig. 3D). Expression analysis confirmed that *Cxcl16* was predominantly expressed by *Adgre1*+ (*F4/80*+) macrophages and cDC2s, while *Cxcr6* was broadly expressed in naïve T cells, CD4+ T cells, CD8+ T cells, and NKT cells (Fig. 2E). In addition, injured PT cells also upregulate *Cxcl16*, albeit at lower levels than macrophages and dendritic cells (Supplemental Fig. 3E), suggesting that tubular cells contribute as an auxiliary source of CXCL16, likely reinforcing T cell recruitment within the inflamed microenvironment. Consistent with these predictions, immunofluorescence staining showed that F4/80+ macrophages were located in close proximity to CXCR6+ cells 14 days after U-IRI (Fig. 2F). To further test the role of macrophages in *Cxcl16* production, we analyzed day 14 U-IRI kidneys from *Ccr2*^−/−^ mice, in which macrophage and dendritic cell recruitment is reduced by 30% and 50%, respectively [28]. Loss of Ccr2 resulted in a 33% reduction in *Cxcl16* expression (Fig. 2G), supporting the hypothesis that recruited macrophages are a major source of *Cxcl16*.

We next sought to define the pathway regulating *Cxcl16* expression in macrophages and injured PT cells. Since IL-1β, TNF-α, and IFN-γ have been reported as CXCL16 inducers [29, 30], we stimulated naïve BMMs and MPT cells with these cytokines. Both IL-1β and/or TNF-α markedly increased *Cxcl16* expression (4–5.6 fold, respectively), whereas IFN-γ, H_2_O_2_, or GM-CSF had no or mild effect (Supplementary Fig. 4). Because IL-1β and TNF-α activate NF-κB, we tested the role of NF-κB signaling by treating BMMs, MPT cells, and primary cultured renal cells (PCRCs) with these cytokines in the presence of the IKKα/IKKβ inhibitor ACHP. IKK inhibition suppressed *Cxcl16* induction by ~32% in BMMs (Fig. 2H), ~50% in MPT cells (Fig. 2J), and 32% in PCRCs (Fig. 2K), respectively. Moreover, BMMs from *Myd88/Trif*-deficient mice, which lack the MyD88 scaffold required for IL-1β/IL-1R1-mediated NF-κB activation [30], failed to upregulate *Cxcl16* in response to IL-1β but responded normally to canonical TNF-α/TNFR1 activation (Fig. 2I); similar results were observed in PCRCs (Fig. 2L).

Together, these results indicate that following kidney injury, recruited macrophages as the predominant source of *Cxcl16*, with injured PT cells contributing as an auxiliary source. Both cell types upregulate *Cxcl16* in response to IL-1β and TNF-α via NF-κB-dependent signaling, facilitating CXCR6+ T cell recruitment to the interstitium and contributing to maladaptive kidney repair.

### CXCR6 promotes T cell accumulation during tubule atrophy

**Fig. 3:**
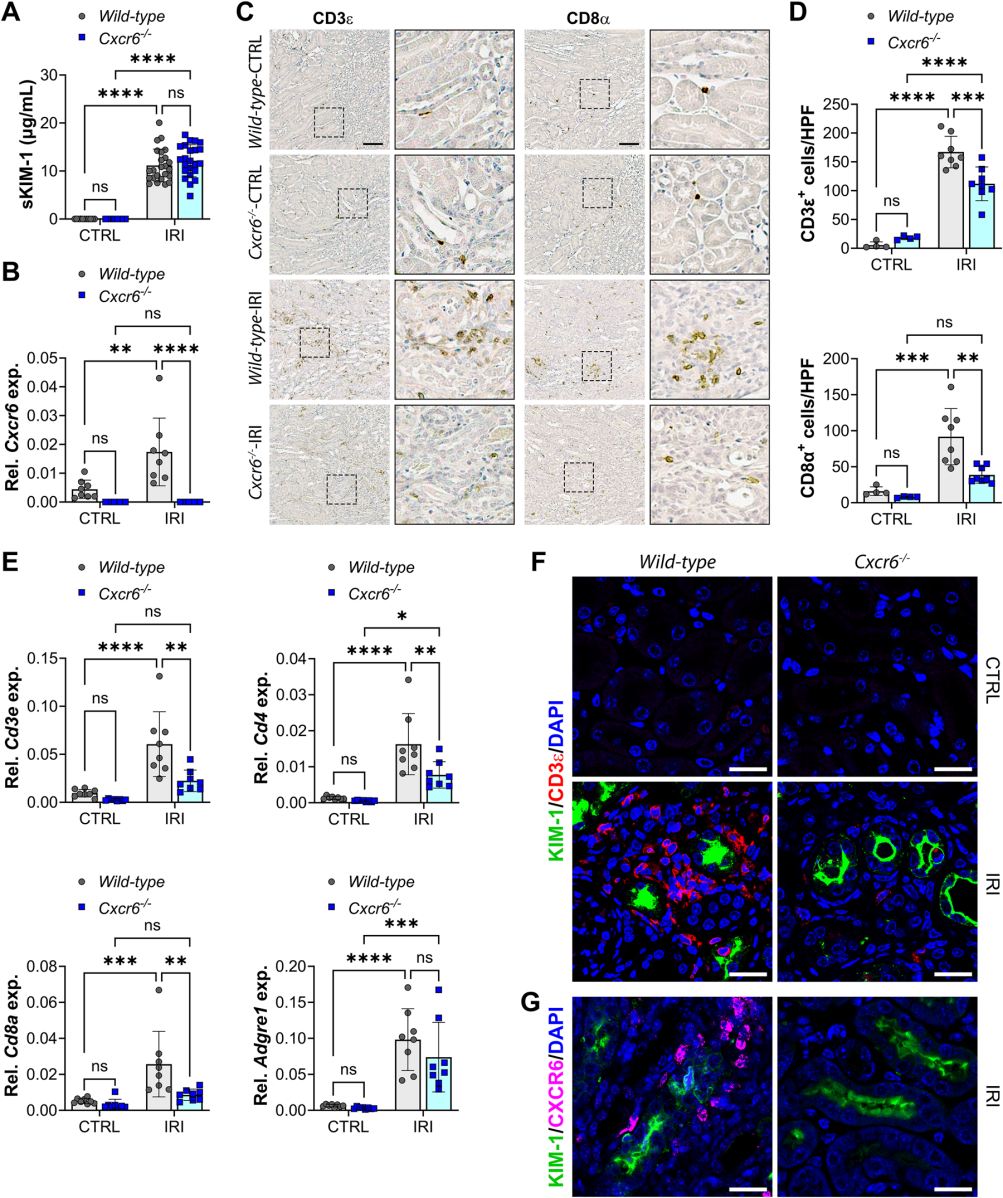
CXCR6 promotes T cell accumulation in late-stage ischemia/reperfusion injury (U-IRI). *Wild-type* and *Cxcr6*^*−/−*^ mice were subjected U-IRI. **A** Serum KIM-1 (sKIM-1) levels of mice were determined by ELISA in control and injured mice on day 1 after U-IRI. *n* = 11–16 mice for baseline/genotype and *n* = 23, 24 mice for IRI/genotype. Two-way ANOVA: *P* = 0.4519 (genotype), *P* < 0.0001 (injury factor), and *P* = 0.4475 (interaction). ****P < 0.0001 by Tukey’s multiple comparison. ns not statistically significant. **B** Healthy control (CTRL) or injured (IRI) kidneys were harvested 14 days after unilateral IRI. Quantitative PCR for *Cxcr6* was performed on whole-kidney mRNA. Two-way ANOVA: *P* < 0.0001 (genotype), *P* = 0.0055 (injury factor), and *P* = 0.0055 (interaction). **P < 0.01 and ****P < 0.0001 by Tukey’s multiple comparison. ns not statistically significant. **C** Kidney sections were immunostained with CD3ε (left panel) and CD8α (right panel) (representative images shown). Scale bars, 100 µm. **D** Quantitation of CD3ε- and CD8α-cell numbers per high power field (HPF), as in (**C**). *n* = 4 CTRL kidneys/genotype and *n* = 8 IRI kidneys/genotype. Two-way ANOVA (injury and genotype interaction): *P* = 0.0036 (CD3ε) and *P* = 0.0442 (CD8α). **P < 0.01, ***P < 0.001, and ****P < 0.0001 by Tukey’s multiple comparison. ns, not statistically significant. **E** Quantitative PCR for *Cd3e*, *Cd4*, *Cd8a*, and *Adgre1* was performed on whole-kidney mRNA. *n* = 8 mice/genotype. Two-way ANOVA (injury and genotype interaction): *P* < 0.0199 (*Cd3e*), *P* = 0.0255 (*Cd4*), *P* = 0.0305 (*Cd8a*), and *P* = 0.3698 (*Adgre1*). *P < 0.05, **P < 0.01, ***P < 0.001, and ****P < 0.0001 by Tukey’s multiple comparison. ns not statistically significant. **F**, **G** Kidney sections were immunofluorescence-stained with KIM-1 (identifies injured PT, green), CD3ε (identifies T cell, red, **F**), CXCR6 (magenta, **G**), and DAPI (identifies nuclei, blue). Original magnification, ×40. Scale bar: 20 μm.

To evaluate the role of CXCL16-CXCR6 signaling in T cell recruitment during AKI-to-CKD transition, we performed U-IRI in *wild-type* and *Cxcr6*^*−/−*^ mice. Serum KIM-1 levels, a marker of acute PT injury, were equally elevated in both groups 1 day post-U-IRI (Fig. 3A), indicating equivalent initial injury. Consistent with the scRNA-seq data, *wild-type* mice showed a significant increase in *Cxcr6* mRNA 14 days after U-IRI, whereas *Cxcr6* expression was undetectable at both time points in kidneys from *Cxcr6*^*−/−*^ mice (Fig. 3B). IHC staining for CD3ε and CD8α demonstrated a decrease in total T cells and CD8α+ cytotoxic T cells in *Cxcr6*^*−/−*^ kidneys on day 14 after U-IRI as compared to *wild-type* kidneys (Fig. 3C, D). Consistent with reduced T cell accumulation, whole-kidney mRNA expression of *Cd3e*, *Cd4*, and *Cd8a* was significantly decreased in *Cxcr6*^*−/−*^ at this time point (Fig. 3E), suggesting that CXCR6 deficiency is associated with reduced recruitment of both CD8α+ and CD4+ T cell populations. In comparison, *Cxcr6* expression was low to absent in *Foxp3*+ regulatory T cell (Treg) and macrophage clusters (Supplementary Fig. 5A). Consistent with this, whole-kidney mRNA expression of canonical Treg markers (*Foxp3*, *Ctla4*, and *Il2ra*) and macrophage marker *Adgre1* (*F4/80*) did not differ significantly *wild-type* and *Cxcr6*^*−/−*^ injured kidneys (Supplementary Fig. 5B and Fig. 3E), consistent with the finding that *Cxcr6* is not expressed on macrophage, monocyte, DC, or Treg populations in the injured kidney (Fig. 2E) [28]. These results indicate that CXCL16-CXCR6 signaling specifically promotes T cell recruitment without affecting macrophage accumulation or Treg-mediated reparative response [31, 32].

### CXCR6 promotes PT cell death via apoptosis and necroptosis

**Fig. 4:**
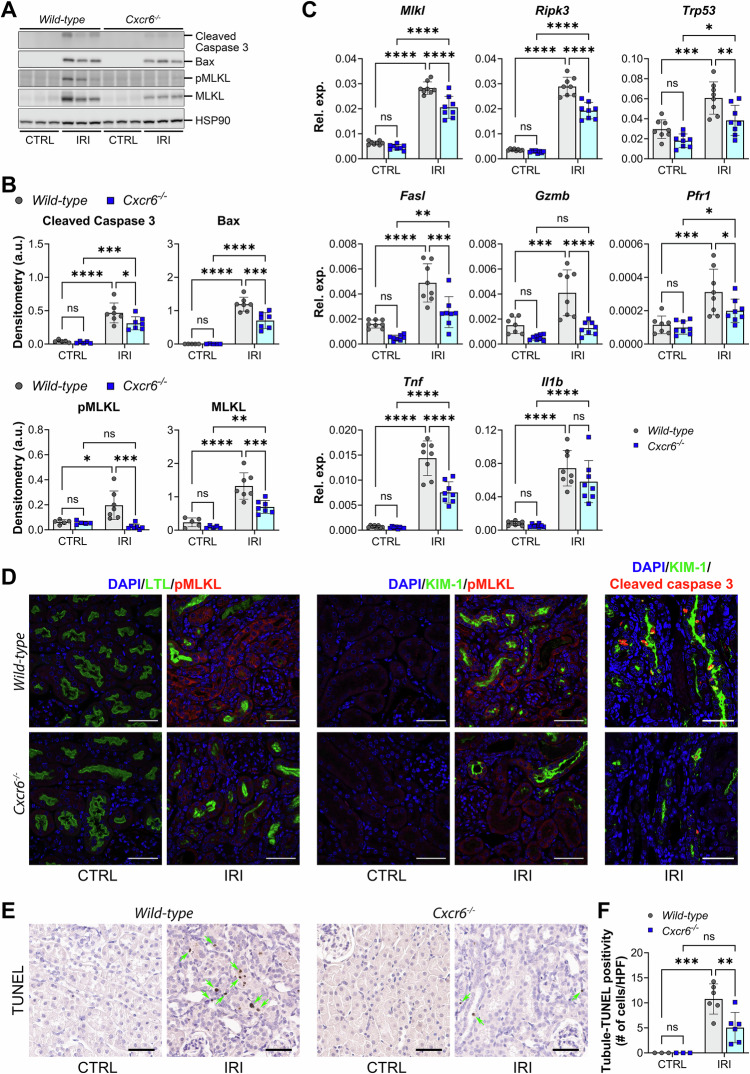
CXCR6 promotes proximal tubule (PT) injury via apoptosis and necroptosis. *Wild-type* and *Cxcr6*^*−/−*^ mice were subjected unilateral ischemia/reperfusion injury (IRI). Healthy control (CTRL) or injured (IRI) kidneys were harvested 14 days after IRI. **A** Western blot analysis of cleaved caspase 3, Bax, phospho-MLKL, MLKL, and HSP90 (loading control, re-probed after stripping) from whole kidney lysates (each lane represents an individual kidney). The full length uncropped original Western blots were provided in Supplementary Fig. 6. **B** Densitometric quantification of protein bands. Expression of cleaved caspase-3, Bax, phospho-MLKL, and MLKL was normalized to HSP90. *n* = 5 CTRL and *n* = 7 IRI kidneys/genotype. Two-way ANOVA (injury and genotype interaction): *P* = 0.1096 (cleaved caspase 3), *P* = 0.0027 (Bax), *P* = 0.0061 (phospho-MLKL), and *P* = 0.0278 (MLKL). *P < 0.05, **P < 0.01, ***P < 0.001, and ****P < 0.0001 by Tukey’s multiple comparison. ns not statistically significant. **C** Quantitative PCR was performed on whole-kidney mRNA. *n* = 8 mice/genotype. Two-way ANOVA (injury and genotype interaction): *P* = 0.0030 (*Mlkl*), *P* < 0.0001 (*Ripk3*), *P* = 0.2288 (*Trp53*), *P* = 0.0943 (*Fasl*), *P* = 0.0187 (*Gzmb*), *P* < 0.0001 (*Tnf*), and *P* = 0.2642 (*Il1b*). *P < 0.05, **P < 0.01, ***P < 0.001, and ****P < 0.0001 by Tukey’s multiple comparison. ns not statistically significant. **D** Kidney sections were immunofluorescence-stained as indicated (representative images shown): LTL (green), pMLKL (red), and DAPI (blue) (left panel); KIM-1 (green), pMLKL (red), and DAPI (blue) (middle panel); and KIM-1 (green), cleaved caspase 3 (red), and DAPI (blue) (right panel). Original magnification, ×40. Scale bar: 50 μm. **E** Kidney sections were immuno-stained with TUNEL (representative images shown). Scale bar: 50 μm. Arrows indicate TUNEL-positive tubular cells. **F** Quantitation of TUNEL-positive tubular cell per high power field (HPF), as in (**E**). *n* = 3 CTRL kidneys/genotype and *n* = 6 IRI kidneys/genotype. Two-way ANOVA (genotype and injury interaction): *P* = 0.0429. **P < 0.01 and ***P < 0.001 by Tukey’s multiple comparison. ns not statistically significant.

In *wild-type* injured kidneys, CD3ε+ and CXCR6+ T cells were frequently localized around KIM-1+ PT, whereas markedly fewer CD3ε+ T cells were observed in *Cxcr6*^*−/−*^ kidneys (Fig. 3E–G). To investigate the functional role(s) of *Cxcr6*-expressing T cells in promoting cell death pathway activation, we assessed markers of apoptosis and necroptosis. The increase in cleaved caspase-3 and Bax protein levels seen in the early phase of tubule atrophy in *wild-type* mice (day 14) was reduced by 23% and 41%, respectively, in *Cxcr6*^*−/−*^ kidneys (Fig. 4A, B). Similarly, *Ripk3*, *Trp53*, and *Mlkl* (mRNA) as well as MLKL and phospho-MLKL (protein) were all reduced on day 14 in the absence of *Cxcr6* as compared to wild-type injured kidneys (Fig. 4A–C). In contrast, one day after injury, T cell markers (*Cd3e*, *Cd4*, and *Cd8a*) were not upregulated in either genotype, and *Cxcr6* deletion had no effect on their expression (Supplementary Fig. 7A). Similarly, phosphorylated MLKL protein levels and necroptosis-related or injury-related transcripts (*Mlkl*, *Ripk3*, and *Havcr1*) were not altered by *Cxcr6* deletion (Supplementary Fig. 7B–D), consistent with unchanged serum KIM-1 levels (Fig. 3A). These data indicate that CXCR6 does not contribute to injury initiation, but rather promotes persistent immune-driven damage during the maladaptive repair phase.

We next examined cytotoxic effector molecules expressed by CD8+ T cells. *Fasl*, *Gmzb*, and *Tnf* were all significantly increased in *wild-type* injured kidneys at day 14 post-U-IRI, but their expression levels were reduced by 48%, 69%, and 48%, respectively in the absence of *Cxcr6* (Fig. 4C). In contrast, the increase in *Il1b*, which is primarily expressed by myeloid cells, was equivalent in *wild-type* and *Cxcr6*^*−/−*^ mice on day 14 after U-IRI, consistent with unchanged *Adgre1* expression (Fig. 4C).

Morphologically, cleaved caspase-3 was primarily localized to a subset of KIM-1-positive PT cells in *wild-type* injured kidneys (Fig. 4D, right panel), while phospho-MLKL was detected at the membranes of KIM-1-positive PT cells and other distal tubular cells (Fig. 4D, middle panel), but was largely absent from LTL-positive “healthy” PT cells in *wild-type* injured kidneys (Fig. 4D, left panel). In *Cxcr6*^*−/−*^ injured kidneys, KIM-1+, cleaved caspase-3+, and phospho-MLKL+ cells appeared less frequently (Fig. 4D). Lastly, tubular TUNEL positivity was significantly reduced in *Cxcr6*^*−/−*^ kidneys compared with *wild-type* kidneys after IRI (Fig. 4E, F), providing quantitative support for reduced cell death in the absence of *Cxcr6*. Together, these findings suggest that CXCR6+ T cells promote both apoptotic and necroptotic PT cell injury during AKI-to-CKD transition.

### Recruitment of CXCR6+ cells impairs functional renal recovery

**Fig. 5:**
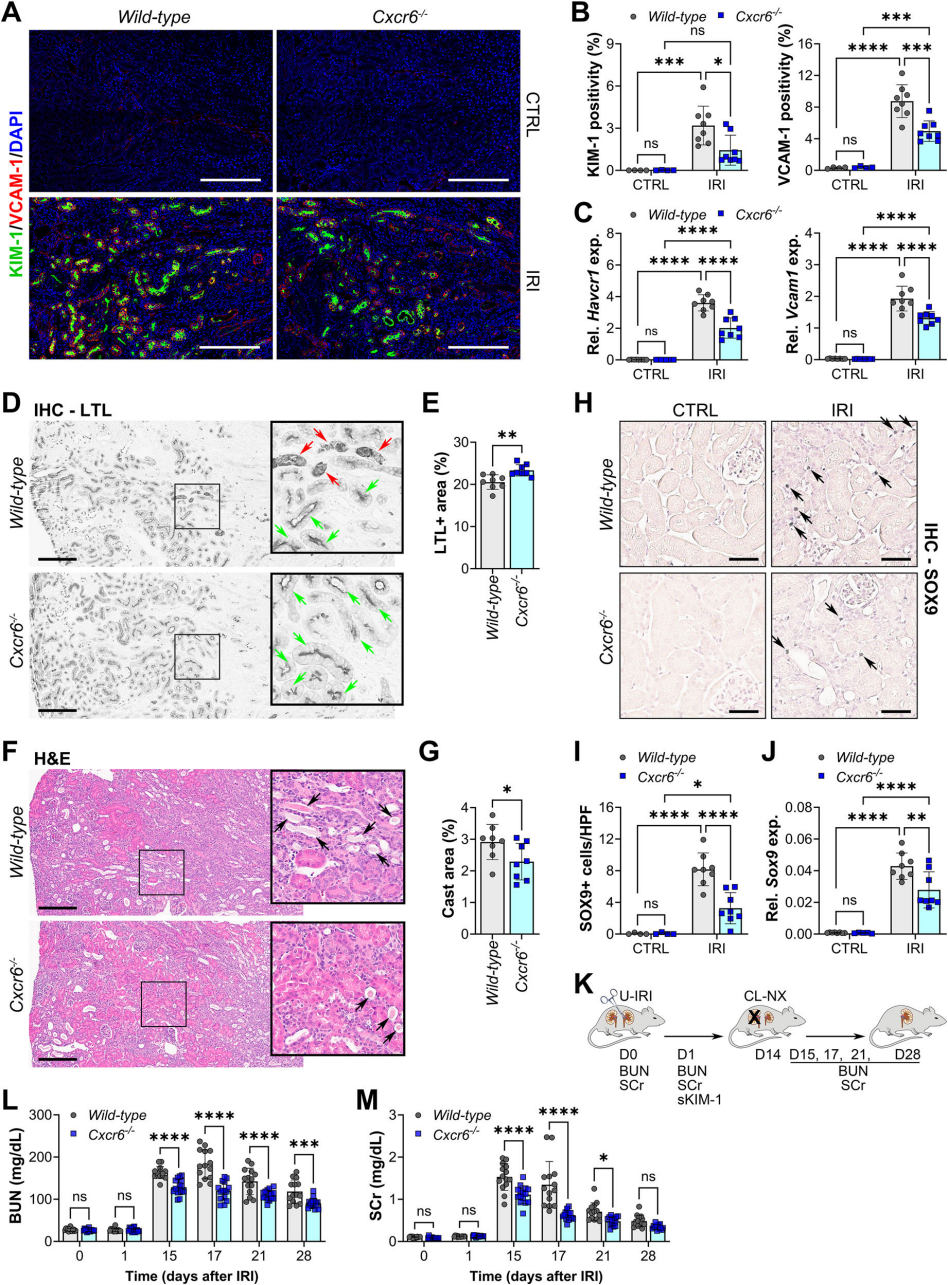
CXCR6 impairs renal function during maladaptive kidney repair. **A**–**D** *Wild-type* and *Cxcr6*^*-/-*^ mice were subjected unilateral ischemia/reperfusion injury (IRI). The injured (IRI) kidneys were harvested 14 days after U-IRI. **A** Kidney sections were immunofluorescence-stained with KIM-1 (green), VCAM-1 (red), and DAPI (blue). Original magnification, ×40. Scale bar: 200 μm. **B** Fluorescence positivity was quantified using ImageJ. *n* = 4 CTRL kidneys/genotype and *n* = 8 IRI kidneys/genotype. Two-way ANOVA (injury and genotype interaction): *P* = 0.0623 (KIM-1) and *P* = 0.0060 (VCAM-1). *P < 0.05, ***P < 0.001, and ****P < 0.0001 by Tukey’s multiple comparison. ns not statistically significant. **C** Quantitative PCR for *Havcr1* and *Vcam1* was performed on whole-kidney mRNA. *n* = 8 mice/genotype. Two-way ANOVA (injury and genotype interaction): *P* < 0.0001 (*Havcr1*) and *P* = 0.0006 (*Vcam1*). ****P < 0.0001 by Tukey’s multiple comparison. ns not statistically significant. **D** Kidney sections were immune-stained with LTL (dark gray) (representative images shown), and LTL positive area was quantified in (**E**). **F** Kidney sections were stained for H&E (representative images shown), and cast area was quantified in (**G**). Green arrows in D indicate LTL-positive tubule. Red arrows in **D** and black arrows in **F** indicate tubular cast. Scale bar: 200 μm. *n* = 8 IRI kidneys/genotype. *P < 0.05 and **P < 0.01 by unpaired t test. **H** Kidney sections were immunohistochemistry-stained for SOX9 (representative images shown). Scale bar: 50 μm. Arrows indicate SOX9-positive tubular cells. **I** Quantitation of SOX9-positive tubular cell per high power field (HPF), as in (**H**). *n* = 4 CTRL kidneys/genotype and *n* = 8 IRI kidneys/genotype. Two-way ANOVA (genotype and injury interaction): *P* = 0.0032. *P < 0.05 and ****P < 0.0001 by Tukey’s multiple comparison. ns not statistically significant. **J** Quantitative PCR for *Sox9* was performed on whole-kidney mRNA. *n* = 8 mice/genotype. Two-way ANOVA (injury and genotype interaction): *P* = 0.0151. **P < 0.01 and ****P < 0.0001 by Tukey’s multiple comparison. ns not statistically significant. **K** Scheme of experimental design. *Wild-type* and *Cxcr6*^*−/−*^ mice were subjected U-IRI, followed by contralateral nephrectomy (CL-NX) on day 14 after U-IRI. The blood was withdrawn for blood urea nitrogen (BUN in **L**) and serum creatinine (SCr in **M**) at the indicated time points. *n* = 14, 15 mice/genotype. Two-way ANOVA (injury and genotype interaction): *P* < 0.0001 (BUN and SCr) by two-way ANOVA. *P < 0.05, ***P < 0.001, and ****P < 0.0001 by Tukey’s multiple comparison. ns not statistically significant.

Compared to *wild-type* kidneys, the expression levels of KIM-1 (*Havcr1*) and VCAM-1 (*Vcam1*) were both significantly lower in *Cxcr6*^*−/−*^ injured kidneys (Fig. 5A–C). In addition, *Cxcr6*^*−/−*^ kidneys exhibited preserved expression of the PT differentiation marker LTL (Fig. 5D, E), decreased cast accumulation (Fig. 5F, G), diminished expression of the dedifferentiation marker SOX9 (Fig. 5H–J), and attenuated interstitial fibrosis on day 14 post-U-IRI (Supplementary Fig. 8). However, the *Cxcr6* null kidneys exhibited no difference in tubular proliferation or kidney weight on day 14 after U-IRI as compared to *wild-type* kidneys (Supplementary Figs. 9 and 10). To determine if the preservation of PT differentiation seen in *Cxcr6*^*−/−*^ kidneys resulted in improved kidney function, we performed contralateral nephrectomy on day 14 post-U-IRI and measured BUN and serum creatinine afterwards (Fig. 5K). One day after nephrectomy (day 15), *Cxcr6*^*−/−*^ mice exhibited significantly less rise in both BUN and serum creatinine demonstrating that the function of the injured kidney was better preserved in these mice (Fig. 5L, M). While GFR slowly improved in both groups over the next 2 weeks, the *Cxcr6*^*−/−*^ mice consistently exhibited lower BUN and creatinine values. These findings indicate that CXCR6+ T cells exacerbate PT injury and reduce the degree of recoverable renal function during the AKI-to-CKD transition.

## Discussion

Our findings highlight a central role for CXCL16-CXCR6 signaling in mediating T cell recruitment during AKI-to-CKD transition. The accumulation of CXCR6+ T cells in the injured kidney suggests that persistent immune activation contributes to prolonged tubular damage, interstitial fibrosis, and impaired renal function. Mechanistically, macrophage-derived CXCL16 serves as a dominant chemotactic signal for CXCR6-expressing T cells, which in turn promote PT injury through cytotoxic mechanisms involving both apoptosis and necroptosis.

Progressive epithelial cell loss is a defining feature of AKI-to-CKD transition and CKD progression, and regulated forms of PT cell death, including necroptosis, ferroptosis, pyroptosis, and apoptosis, have emerged as central drivers of maladaptive repair. Inhibition of necroptosis improves renal outcomes in subtotal nephrectomy and unilateral ureter obstruction (UUO) models [33, 34]. Inhibition of ferroptosis using liproxstatin and pyroptosis using VX-765 reduces the inflammatory response and interstitial fibrosis and ameliorates renal function 14 days after bilateral IRI [5]. On the other hand, apoptosis can also promote loss of renal epithelial cells during chronic tubular atrophy, which can be activated by FasL/Fas/Bax-mediated mitochondrial injury, ER stress, aminoglycosides, and cisplatin [35]. Single-cell regulatory network inference and clustering (SCENIC) analysis of IRI kidneys revealed increased regulon activity of Myc in the *Vcam1+/Ccl2+* “late injured” PT cluster, a factor known to be involved in tubular cell apoptosis [36]. We now show that injured PTs exhibit elevated apoptotic and necroptotic signaling, evidenced by increased cleaved caspase-3, Bax, MLKL, and phospho-MLKL expression. Although MLKL phosphorylation is not sufficient to definitively establish execution of necroptosis, its presence supports activation of necroptosis-associated signaling in these cells [37], suggesting that necroptotic cell death may occur in a subset of phosphorylated MLKL-positive tubules.

Necroptosis-driven necroinflammation has emerged as a key mechanism linking unresolved AKI to chronic inflammation and fibrosis. Unlike apoptosis, necroptosis culminates in MLKL-mediated plasma membrane disruption, resulting in the release of damage-associated molecular patterns (DAMPs) that amplify innate immune signaling [38–40]. In mouse models of IRI, RIPK3-MLKL-dependent necroptosis promotes inflammasome activation, macrophage recruitment, and long-term interstitial fibrosis [4]. In our U-IRI model, MLKL and phospho-MLKL induction, predominantly within injured PT cells, was markedly attenuated in *Cxcr6*^*−/−*^ kidneys, accompanied by reduced VCAM-1 expression and improved structural and functional outcomes. These findings suggest that CXCR6-dependent immune cell recruitment sustains a necroinflammatory microenvironment that perpetuates tubular injury and fibrotic remodeling. Persistent necroptotic signaling within injured tubules may reinforce immune cell retention and promote fibroblast or myofibroblast activation through paracrine inflammatory cues, forming a feed-forward loop of maladaptive repair. Disruption of this loop in *Cxcr6*-deficient mice appears to mitigate progression from AKI to CKD. Although the specific pathways through which CXCR6+ T cells promote necroptosis remain to be fully defined, our data suggest that this effect is likely mediated indirectly via cytokine- and death receptor-dependent signaling, rather than through direct enzymatic activity.

Upstream of this process, NF-κB-dependent activation of macrophages plays a pivotal role in shaping the inflammatory milieu of the injured kidney [41]. We demonstrate that IL-1β and TNF-α induce CXCL16 expression predominantly in macrophages, with injured PT cells also contributing via NF-κB-dependent signaling, thereby linking innate immune activation within both immune and parenchymal compartments to adaptive immune recruitment. Consistent with this model, *Cxcr6* deletion reduced expression of cytotoxic effector molecules, including *Fasl*, *Gzmb*, and *Tnf*, implicating CXCR6+ T cells as amplifiers of tubular cytotoxicity. Prior studies have shown that CXCR6+ T cells migrate to injured kidneys and promote fibrosis through production of IL-17A and PD-1 signaling in models of bilateral IRI, UUO, and DOCA/salt hypertension [42, 43]. Although direct pharmacologic targeting of CXCR6 remains challenging, our data highlight NF-κB-CXCL16 signaling in macrophages and injured tubular cells as a tractable upstream axis to limit pathogenic T cell recruitment and immune-mediated tubular injury.

Beyond CD8α+ T cells, CXCL16-CXCR6 signaling also contributes to the recruitment of CD4+ T cell populations. While CD8α T cells are classically associated with direct cytotoxicity toward injured tubular cells, CD4 T cells may contribute indirectly by sustaining inflammatory signaling, enhancing antigen presentation, and promoting profibrotic immune responses. T helper 17 (Th17) cells, in particular, exacerbate renal inflammation and injury [7], whereas Tregs facilitate injury resolution and tissue repair [31, 32]. Notably, *Cxcr6* deletion reduced recruitment of conventional CD4+ T cells without affecting Treg abundance, potentially shifting the immune balance toward a reparative phenotype. This selective modulation of pathogenic, but not protective, T cell subsets may contribute to the improved structural and functional recovery observed in *Cxcr6*-deficient kidneys.

In conclusion, our study establishes macrophage-derived CXCL16 as the predominant chemokine driving recruitment of CXCR6+ T cells during the AKI-to-CKD transition, with injured PT cells providing an additional local source of CXCL16 within the inflamed microenvironment. Accumulation of CXCR6+ T cells promotes sustained apoptotic and necroptotic signaling in injured tubules, exacerbating fibrosis and limiting renal recovery. Targeting the CXCL16-CXCR6 axis, or its upstream inflammatory regulators, represents a promising strategy to disrupt maladaptive immune-epithelial crosstalk and preserve kidney function, warranting further investigation in human CKD.

## Supplementary information

Original Data

Supplemental Material

## Data Availability

The scRNA-seq dataset analyzed in this study is available in the Gene Expression Omnibus (GEO) under accession number GSE197626. The data that support the findings of this study are available from the corresponding author upon reasonable request.
