# Supplementary material for: CXCR6+ T cells promote apoptosis and necroptosis in proximal tubules during AKI-to-CKD transition
Source: Cell Death Dis. 2026 Mar 24;17(1):359. doi: 10.1038/s41419-026-08644-x (PMC13039913; doi:10.1038/s41419-026-08644-x)
Supplement: Supplementary file 1 — Original Data [file 41419_2026_8644_MOESM1_ESM.pptx]

## Slide 1
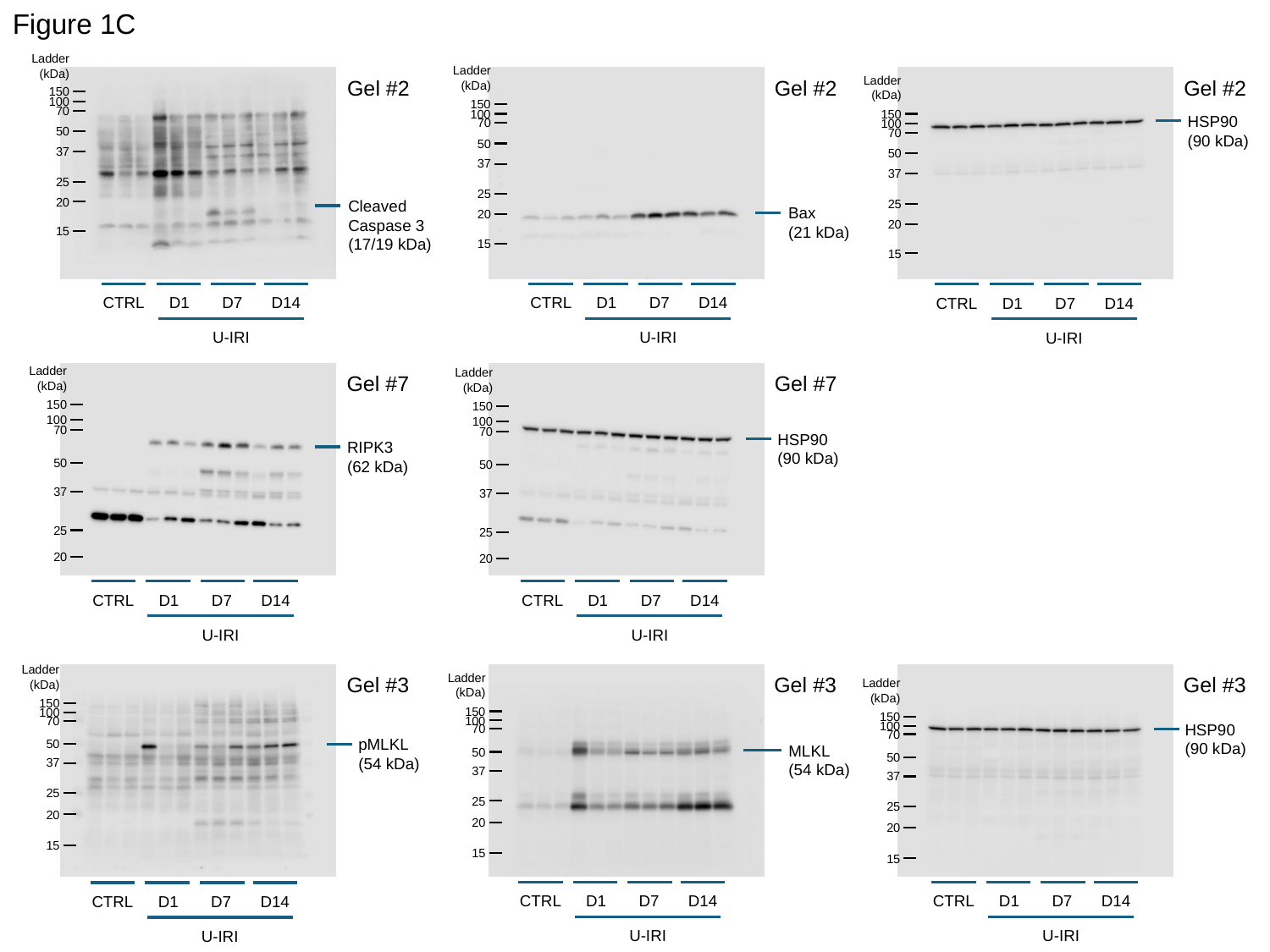

Figure 1C
Ladder
(kDa)
Ladder
(kDa)
Ladder
(kDa)
Gel #2
Gel #2
Gel #2
150
100
150
70
100
150
HSP90
(90 kDa)
70
100
50
70
50
37
50
37
37
25
25
20
Cleaved
Caspase 3
(17/19 kDa)
25
Bax
(21 kDa)
20
20
15
15
15
CTRL
D1
D7
D14
U-IRI
CTRL
D1
D7
D14
U-IRI
CTRL
D1
D7
D14
U-IRI
Ladder
(kDa)
Ladder
(kDa)
Gel #7
Gel #7
150
150
100
100
70
70
HSP90
(90 kDa)
RIPK3
(62 kDa)
50
50
37
37
25
25
20
20
CTRL
D1
D7
D14
U-IRI
CTRL
D1
D7
D14
U-IRI
Ladder
(kDa)
Ladder
(kDa)
Gel #3
Gel #3
Gel #3
Ladder
(kDa)
150
150
100
150
100
70
100
HSP90
(90 kDa)
70
70
pMLKL
(54 kDa)
50
MLKL
(54 kDa)
50
50
37
37
37
25
25
25
20
20
20
15
15
15
CTRL
D1
D7
D14
U-IRI
CTRL
D1
D7
D14
U-IRI
CTRL
D1
D7
D14
U-IRI

## Slide 2
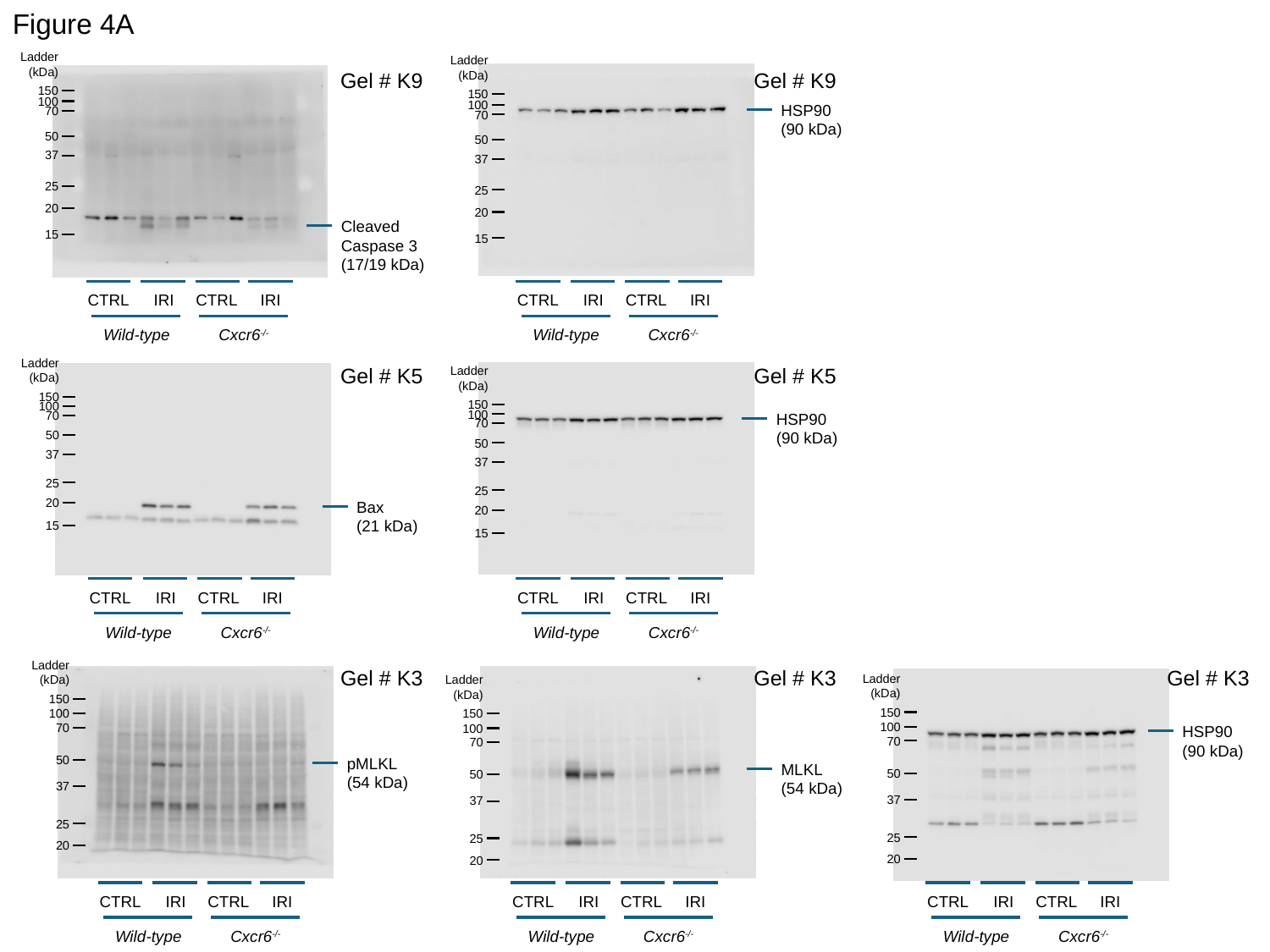

Figure 4A
Ladder
(kDa)
150
100
70
50
37
25
20
Cleaved
Caspase 3
(17/19 kDa)
15
CTRL
IRI
CTRL
IRI
Wild-type Cxcr6-/-
Ladder
(kDa)
150
100
HSP90
(90 kDa)
70
50
37
25
20
15
CTRL
IRI
CTRL
IRI
Wild-type Cxcr6-/-
Gel # K9
Gel # K9
Ladder
(kDa)
150
100
70
50
37
25
20
Bax
(21 kDa)
15
CTRL
IRI
CTRL
IRI
Wild-type Cxcr6-/-
Gel # K5
Gel # K5
Ladder
(kDa)
150
100
HSP90
(90 kDa)
70
50
37
25
20
15
CTRL
IRI
CTRL
IRI
Wild-type Cxcr6-/-
Ladder
(kDa)
150
100
70
50
pMLKL
(54 kDa)
37
25
20
CTRL
IRI
CTRL
IRI
Wild-type Cxcr6-/-
Gel # K3
Gel # K3
Gel # K3
Ladder
(kDa)
150
100
HSP90
(90 kDa)
70
50
37
25
20
CTRL
IRI
CTRL
IRI
Wild-type Cxcr6-/-
Ladder
(kDa)
150
100
70
MLKL
(54 kDa)
50
37
25
20
CTRL
IRI
CTRL
IRI
Wild-type Cxcr6-/-

## Slide 3
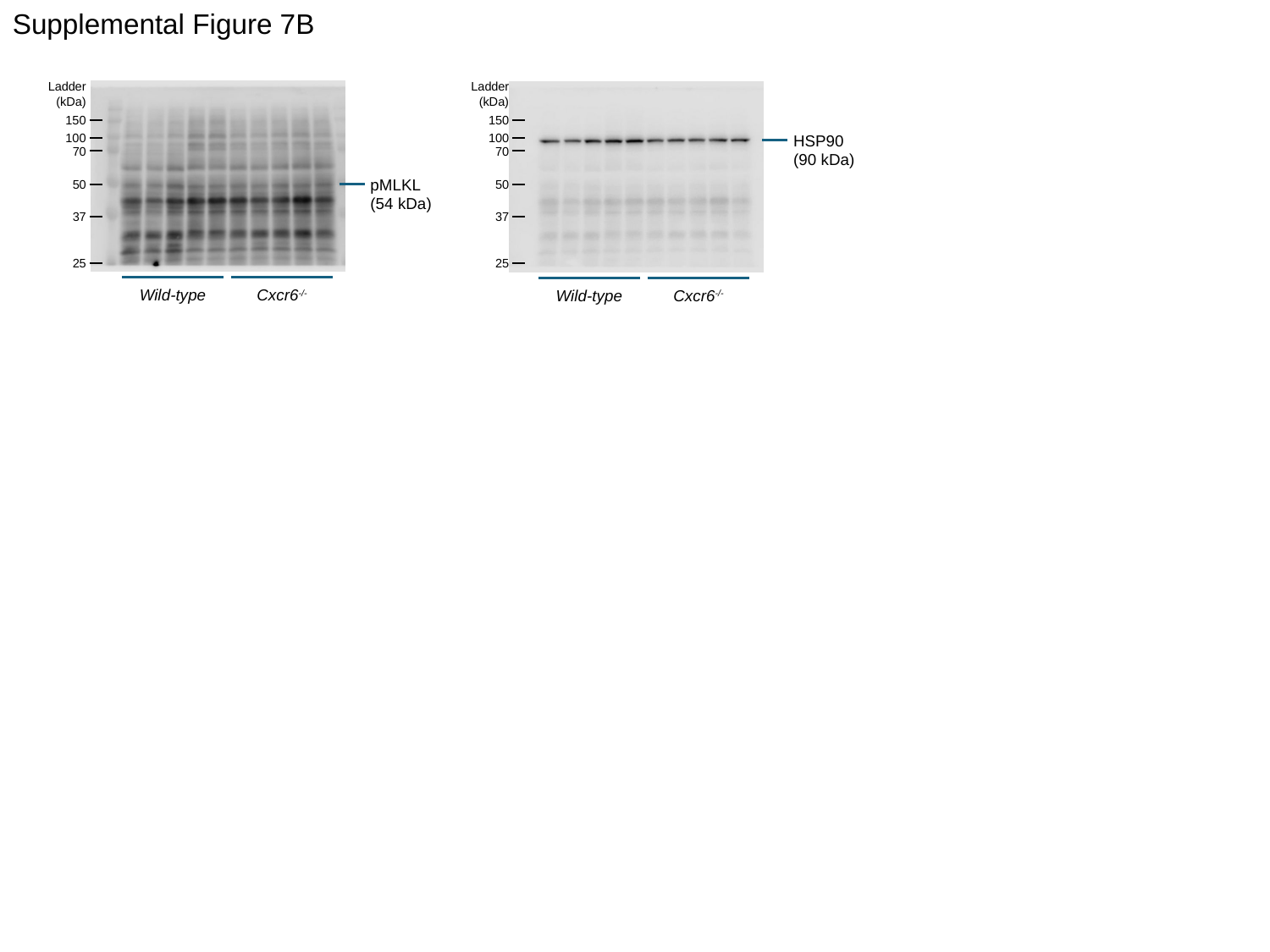

Supplemental Figure 7B
Ladder
(kDa)
Ladder
(kDa)
150
150
100
100
HSP90
(90 kDa)
70
70
pMLKL
(54 kDa)
50
50
37
37
25
25
Wild-type
Cxcr6-/-
Wild-type
Cxcr6-/-
