# Supplementary material for: CXCR6+ T cells promote apoptosis and necroptosis in proximal tubules during AKI-to-CKD transition
Source: Cell Death Dis. 2026 Mar 24;17(1):359. doi: 10.1038/s41419-026-08644-x (PMC13039913; doi:10.1038/s41419-026-08644-x)
Supplement: Supplementary file 2 — Supplemental Material [file 41419_2026_8644_MOESM2_ESM.pdf]

Supplementary Material

CXCR6+ T Cells Promote Apoptosis and Necroptosis in Proximal Tubules During AKI-to-CKD Transition

Xiaoxu Li[1], Isabel Melchinger[1], Yuchu Chen[1], Jiankan Guo[1], Lloyd G Cantley[1], Leyuan Xu[1,*]

[1]Department of Internal Medicine/Section of Nephrology, Yale University School of Medicine

Corresponding author:
Leyuan Xu, Ph.D.
Department of Internal Medicine/Section of Nephrology
Yale University School of Medicine
PO Box 208029
New Haven, CT 06520

Phone: (203) 785-7111
Fax: (203) 785-7068

Email: leyuan.xu@yale.edu

Supplemental Tables:

Primer sequences to perform qPCR.

Gene	Forward	Reverse
Adgre1	TGAATGGCTCCATTTGTGAA	GATGGCCAAGGATCTGAAAA
Cd3e	GAAAGCTCGAGTGTGTGAGT	GCCTTGGCCTTCCTATTCTT
Cd4	GAGAGTTCCCAGAAGAAGATCAC	AGGCGAACCTCCTCTAATTAATAC
Cd8a	GTGGACCTGGTATGTGAAGTG	TGAAGCCATATAGACAACGAAGG
Col1a1	GAAACCCGAGGTATGCTTGA	GGGTCCCTCGACTCCTACAT
Ctla4	CTCTGAAGCCATACAGGTGAC	AATCTAGGAAGCCCACTGTATTC
Cxcl16	TGTCCATTCTTTATCAGGTTCCA	AACTCTTCCCATGACCAGTTC
Cxcr6	CTGGGCTTCTCTTCTGATGC	CGTTTGTTCTCCTGGCTGTTA
Fasl	TGGCCCATTTAACAGGGAAC	CAACCTCTTCTCCTCCATTAGC
Foxp3	GCAATAGTTCCTTCCCAGAGTT	GTAGGCGAACATGCGAGTAA
Gzmb	CTGCTCACTGTGAAGGAAGTATAA	TCTAGTCCTCTTGGCCTTACTC
Havcr1	GAGAGTGACAGTGGTCTGTATTG	CGTGTGGGAATCTCTGGTTTA
Hprt1	CAGTACAGCCCCAAAATGGT	CAAGGGCATATCCAACAACA
Il1b	GAGGACATGAGCACCTTCTTT	CATGGAGAATATCACTTGTTGGTTG
Il2ra	TCTACAGAGAGGTCCTGCTATT	CTGGCCACTGCTACCTTATAC
Mlkl	TGAGGGAACTGCTGGATAGA	CCGAATGGTGTAGCCTGTATAA
Prf1	TTGGTGGGACTTCAGCTTTC	CATACACCTGGCACGAACTT
Ripk3	GCACTCCTCAGATTCCACATAC	GTGTCTTCCATCTCCCTGATTC
Sox9	GGAACAGACTCACATCTCTCCTAATG	CTGAGATTGCCCAGAGTGCT
Tnf	AGACCCTCACACTCAGATCA	AAGAGAACCTGGGAGTAGACA
Trp53	TCTGTTATGTGCACGTACTCTC	ATTTCCTTCCACCCGGATAAG
Vcam1	ACTCCCGTCATTGAGGATATTG	GTTGTATTCCTGGGAGAGATGTAG

Supplemental Figures:

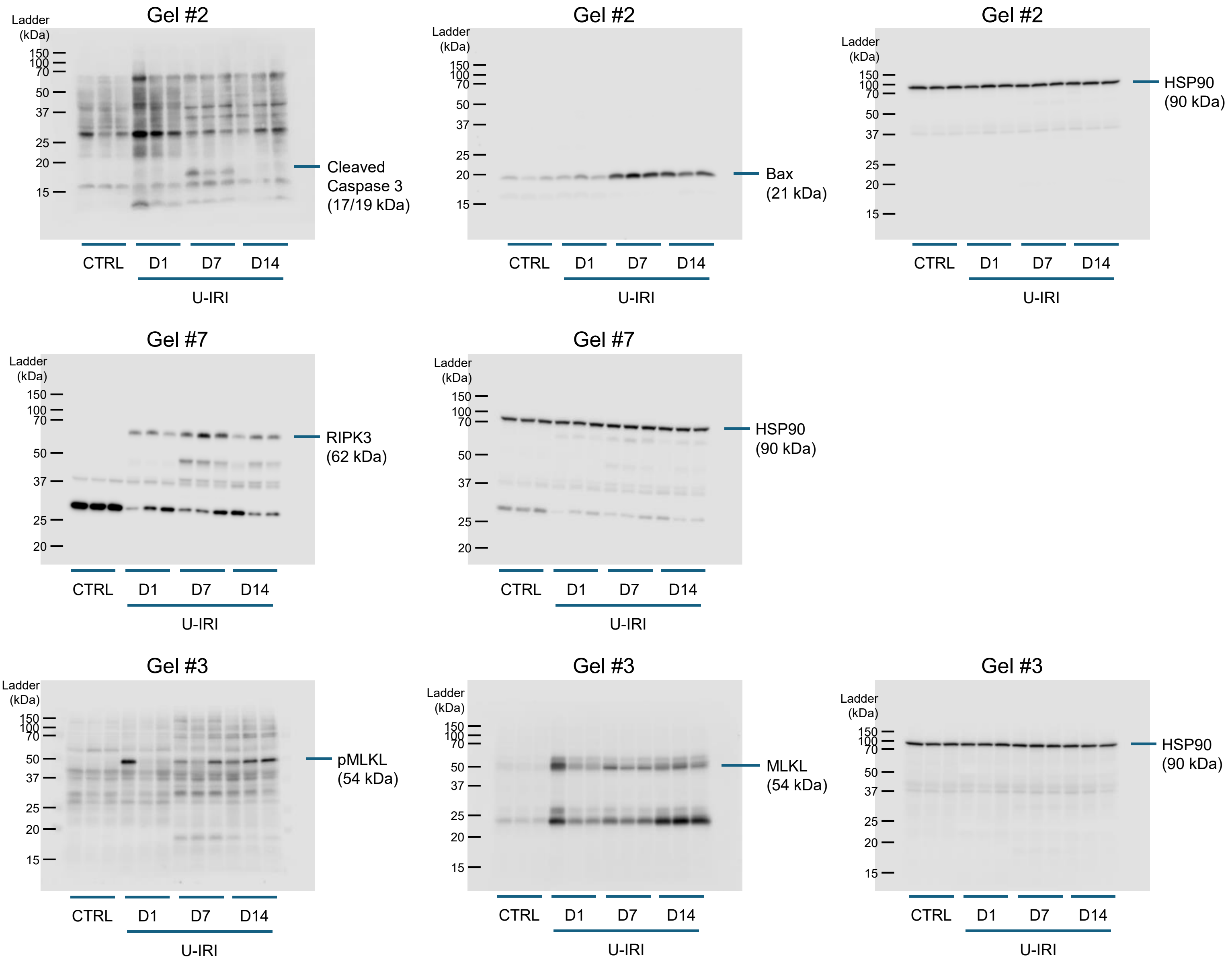

Supplemental Figure 1. The full length uncropped original Western blots.

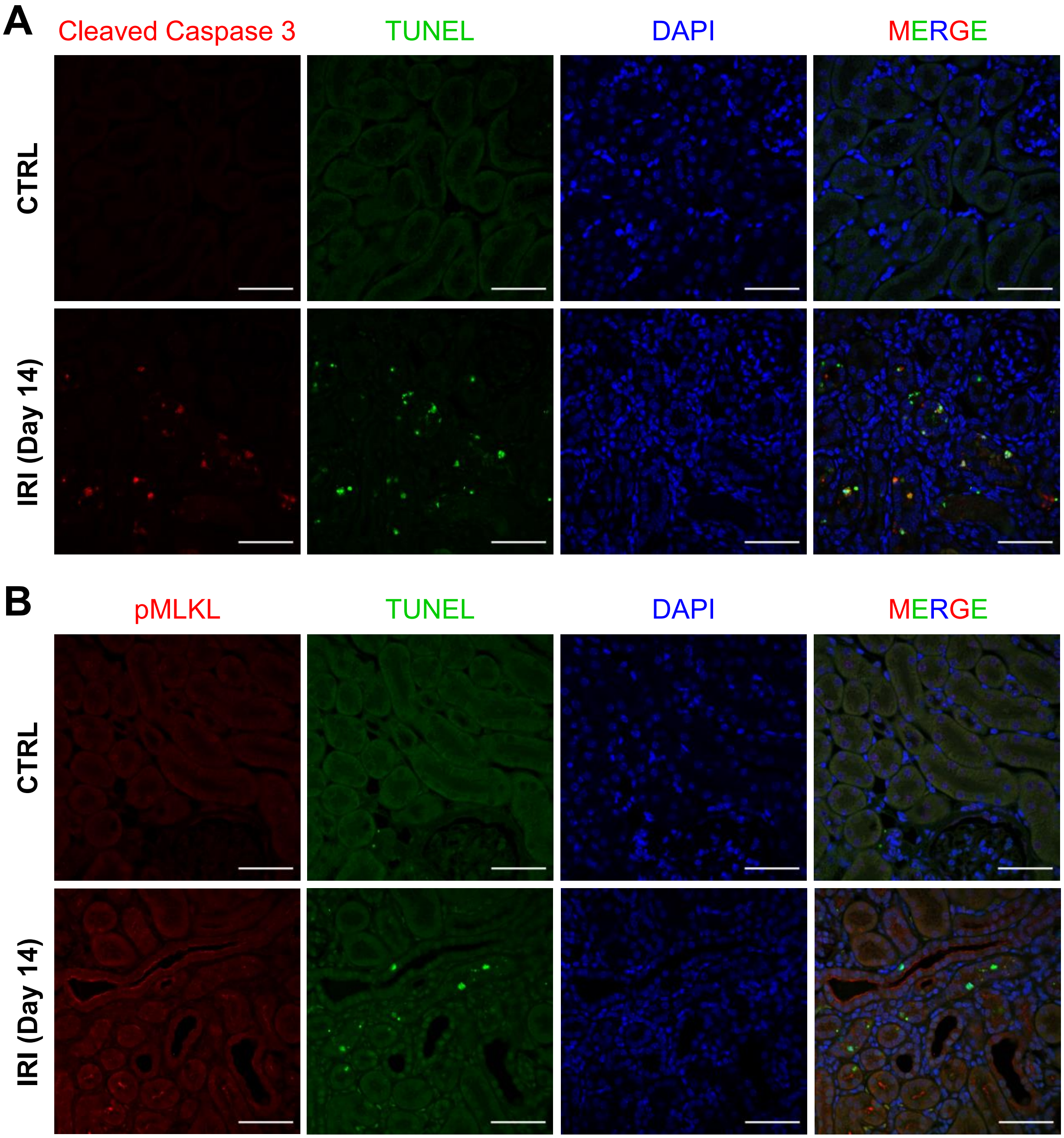

Supplemental Figure 2. Apoptotic and necroptotic cell death in injured kidneys during AKI-to-CKD transition. *Wild-type* mice were subjected to unilateral ischemia/reperfusion injury (U-IRI), and the injured kidneys were harvested on day 14 post-injury. Control (CTRL) kidneys were obtained from healthy uninjured mice. Kidney sections were immunofluorescence-stained for cleaved caspase 3 (A), phosphorylated MLKL (pMLKL; B), TUNEL, and DAPI. Original magnification, ×63. Scale bar: 50 μm.

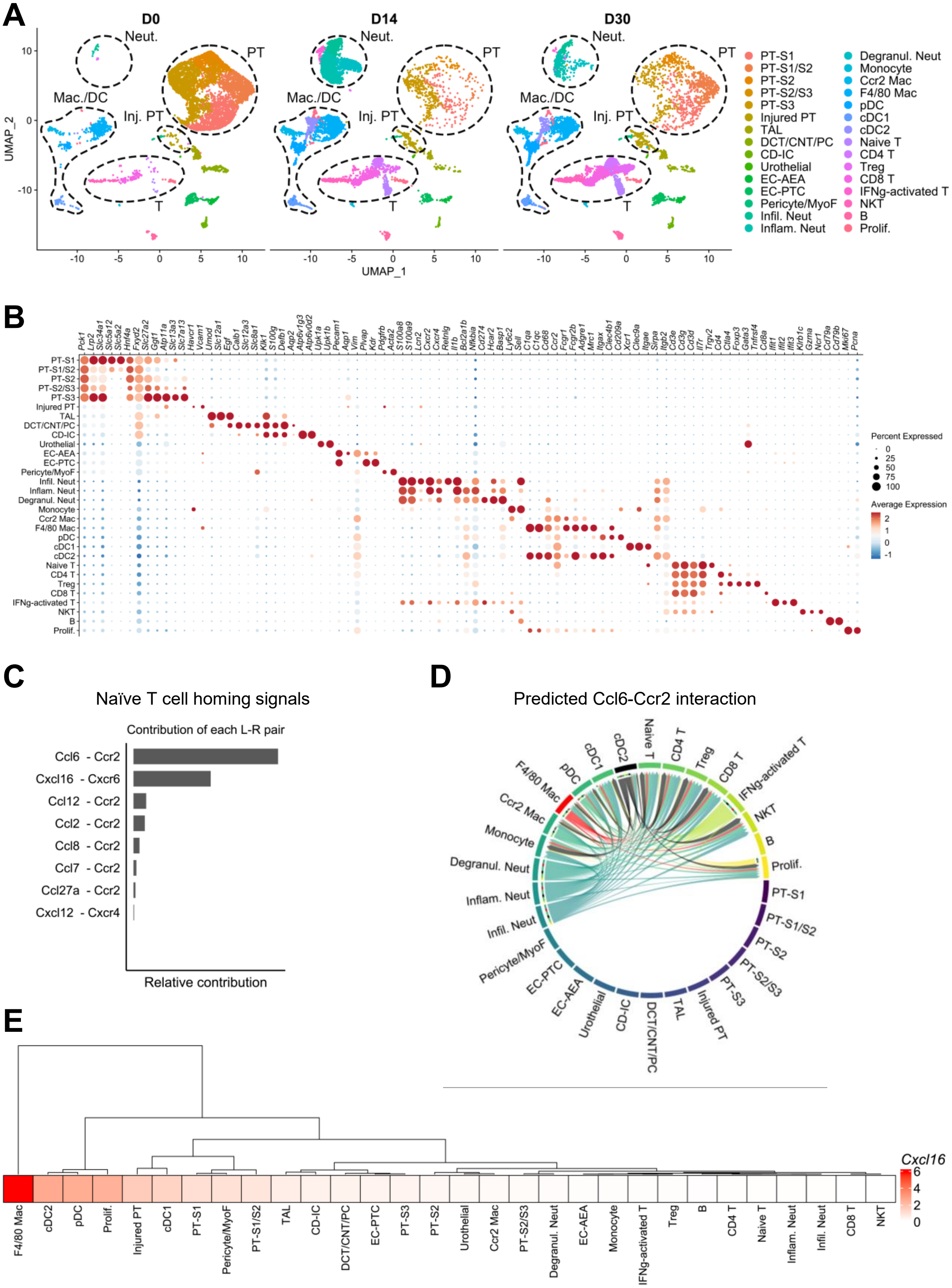
A
D0
D14
D30
Neut.
PT
Mac./DC
Inj. PT
T
UMAP_2
UMAP_1
PT-S1
PT-S1/S2
PT-S2
PT-S2/S3
PT-S3
Injured PT
TAL
DCT/CNT/PC
CD-IC
Urothelial
EC-AEA
EC-PTC
Pericyte/MyoF
Infil. Neut
Inflam. Neut
Degranul. Neut
Monocyte
Ccr2 Mac
F4/80 Mac
pDC
cDC1
cDC2
Naive T
CD4 T
Treg
CD8 T
IFNg-activated T
NKT
B
Prolif.
B
Percent Expressed
Average Expression
C
Naïve T cell homing signals
Contribution of each L-R pair
Ccl6 - Ccr2
Cxcl16 - Cxcr6
Ccl12 - Ccr2
Ccl2 - Ccr2
Ccl8 - Ccr2
Ccl7 - Ccr2
Ccl27a - Ccr2
Cxcl12 - Cxcr4
Relative contribution
D
Predicted Ccl6-Ccr2 interaction
E
Cxcl16

Supplemental Figure 3. Identification of T cell homing signals during AKI-to-CKD transition. *Wild-type* mice were subjected to unilateral ischemia/reperfusion injury (U-IRI), and the injured kidneys were harvested on day 14 and 30 post-injury for single-cell RNA sequencing analysis as previously reported (GSE197626)[1]. (A) An integrated uniform manifold approximation and projection (UMAP) of 30,323 cells from IRI and control healthy kidneys was split into 3 sub-datasets. Cell clusters were identified using the integrated data from all cells by kidney cell and immune cell lineage-specific marker expression, as shown in B. Ligand-receptor interaction was analyzed using CellChat R package. (C) The contribution of each ligand-receptor pair to the overall signaling pathway (CCL and CXCL) in naïve T cells was computed and visualized in a bar graph. (D) The cell-cell communication mediated by *Ccl6*-*Ccr2* pair was visualized in a chord hierarchy plot. PT, proximal tubule; TAL, thick ascending limb; DCT, distal convoluted tubule; CNT, connecting tubule; PC, principal cell; CD-IC, collecting duct-intercalated cell; EC-AEA, endothelia cell-afferent/efferent arteriole; EC-PTC, peritubular endothelia cell; MyoF, myofibroblast; Infil. Neut, infiltrating neutrophil; Inflam. Neut, inflammed neutrophil; Degranul. Neut, degranulated neutrophil; Mac, macrophage; pDC, plasmacytoid dendritic cell; cDC, conventional dendritic cell; T, T cells; NK, natural killer cells; B, B cells; Prolif, proliferating cells. (E) Average *Cxcl16* expression across all cell types in injured kidneys was visualized as a heatmap.

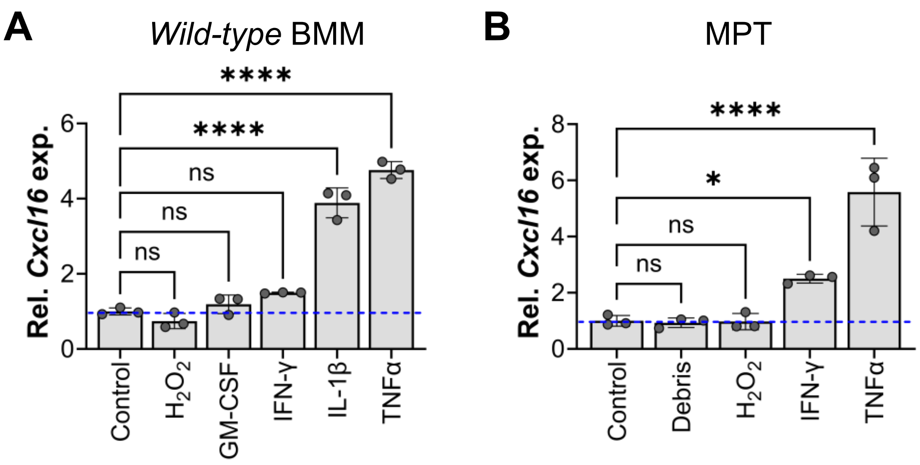

Supplemental Figure 4. Inflammatory cytokines induce *Cxcl16* expression in macrophage and tubular cell in vitro. (A) *Wild-type* bone marrow-derived macrophages (BMDMs) were treated with PBS (control), H_2O_2 (500 μM), granulocyte-macrophage colony-stimulating factor (GM-CSF, 25 ng/mL), interferon γ (IFN-γ, 100 ng/mL), interleukin-1β (IL-1β, 10 ng/mL), or tumor necrosis factor-α (TNF-α) (20 ng/mL) for 6 hrs. (B) Mouse proximal tubule (MPT) cells were treated with PBS (control), cell debris, H_2O_2 (500 μM), IFN-γ (100 ng/mL), or TNF-α (20 ng/mL) for 6 hrs. Quantitative PCR for *Cxcl16* was performed on BMDM and MPT mRNA. n=3 independently treated experiments. $P<0.0001$ by one-way ANOVA. *$P<0.05$ and ****$P<0.0001$ by Tukey's multiple comparison. ns, not statistically significant.

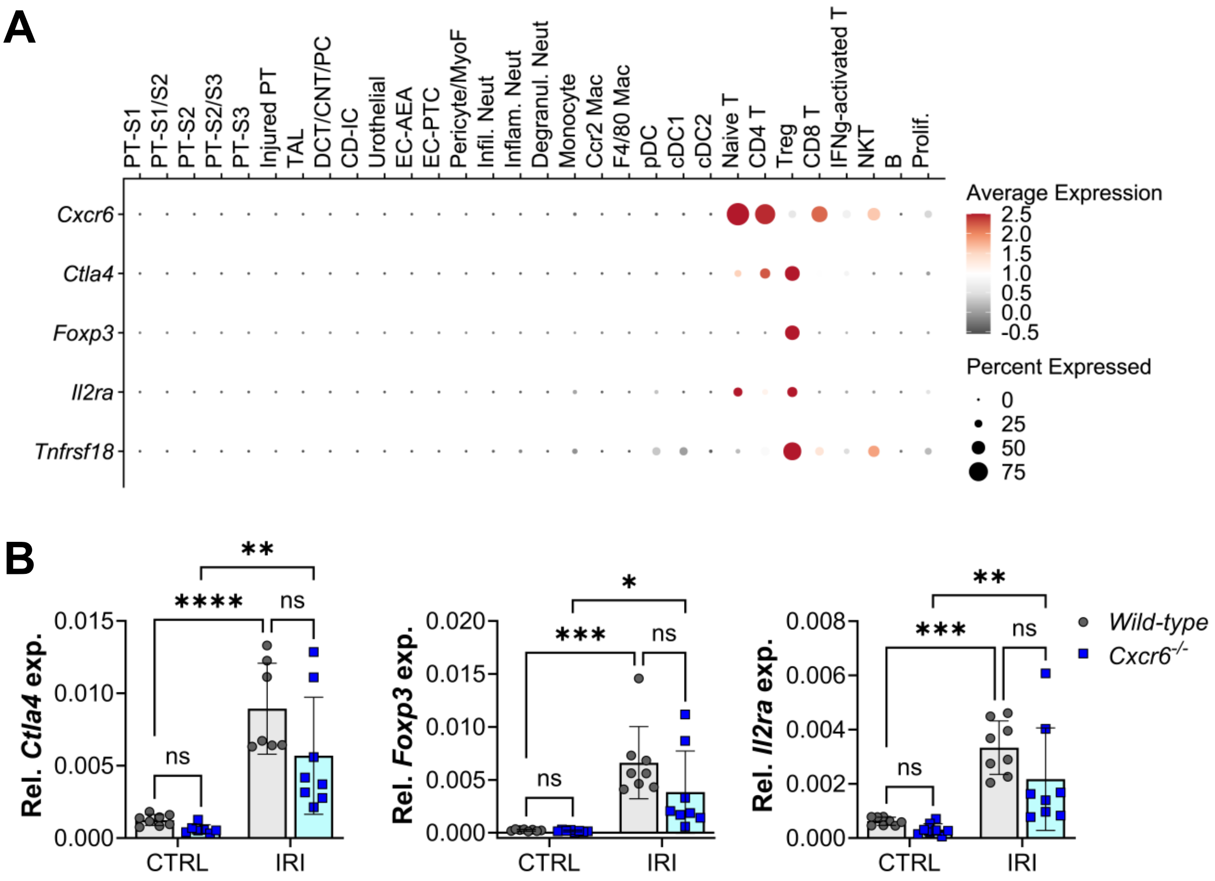

Supplemental Figure 5. CXCR6 deficiency does not alter regulatory T (Treg) cell accumulation in late-stage ischemia/reperfusion injury (U-IRI). (A) Expression of *Cxcr6* and canonical Treg markers (*Ctla4*, *Foxp3*, *Il2ra*, and *Tnfrsf18*) was visualized in a dot plot using the scRNA-seq dataset shown in Supplemental Figure 3A-B. (B) *Wild-type* and *Cxcr6*$^{-/-}$ mice were subjected U-IRI. Healthy control (CTRL) or injured (IRI) kidneys were harvested 14 days after U-IRI. Quantitative PCR for *Ctla4*, *Foxp3*, and *Il2ra* was performed on whole-kidney mRNA. Two-way ANOVA revealed no significant interaction between genotype and injury: P=0.1797 (*Ctla4*), P=0.1499 (*Foxp3*), and P=0.2682 (*Il2ra*). *P<0.05, **P<0.01, ***P<0.001, and ****P<0.0001 by Tukey's multiple comparison. ns, not statistically significant.

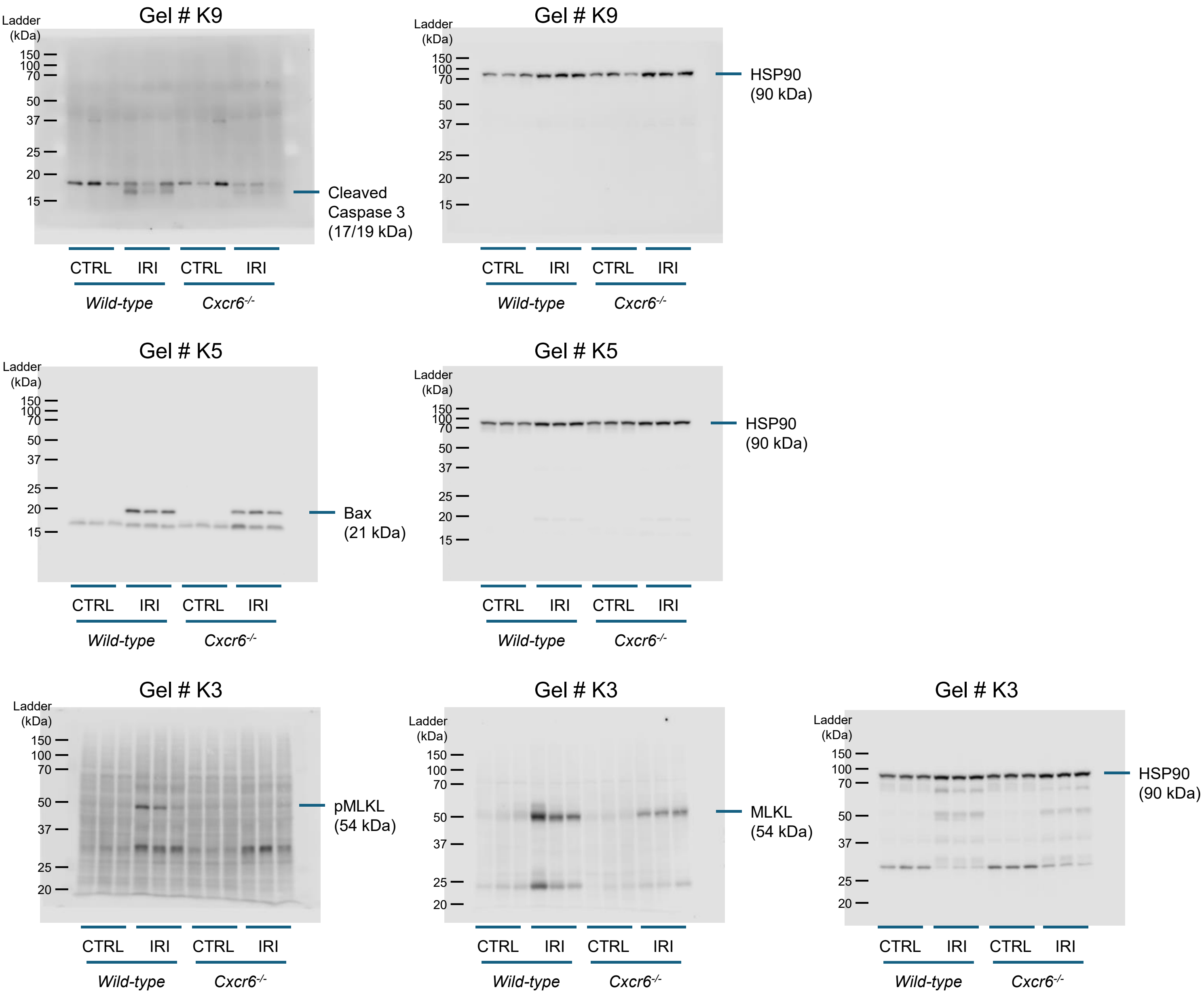

Supplemental Figure 6. The full length uncropped original Western blots.

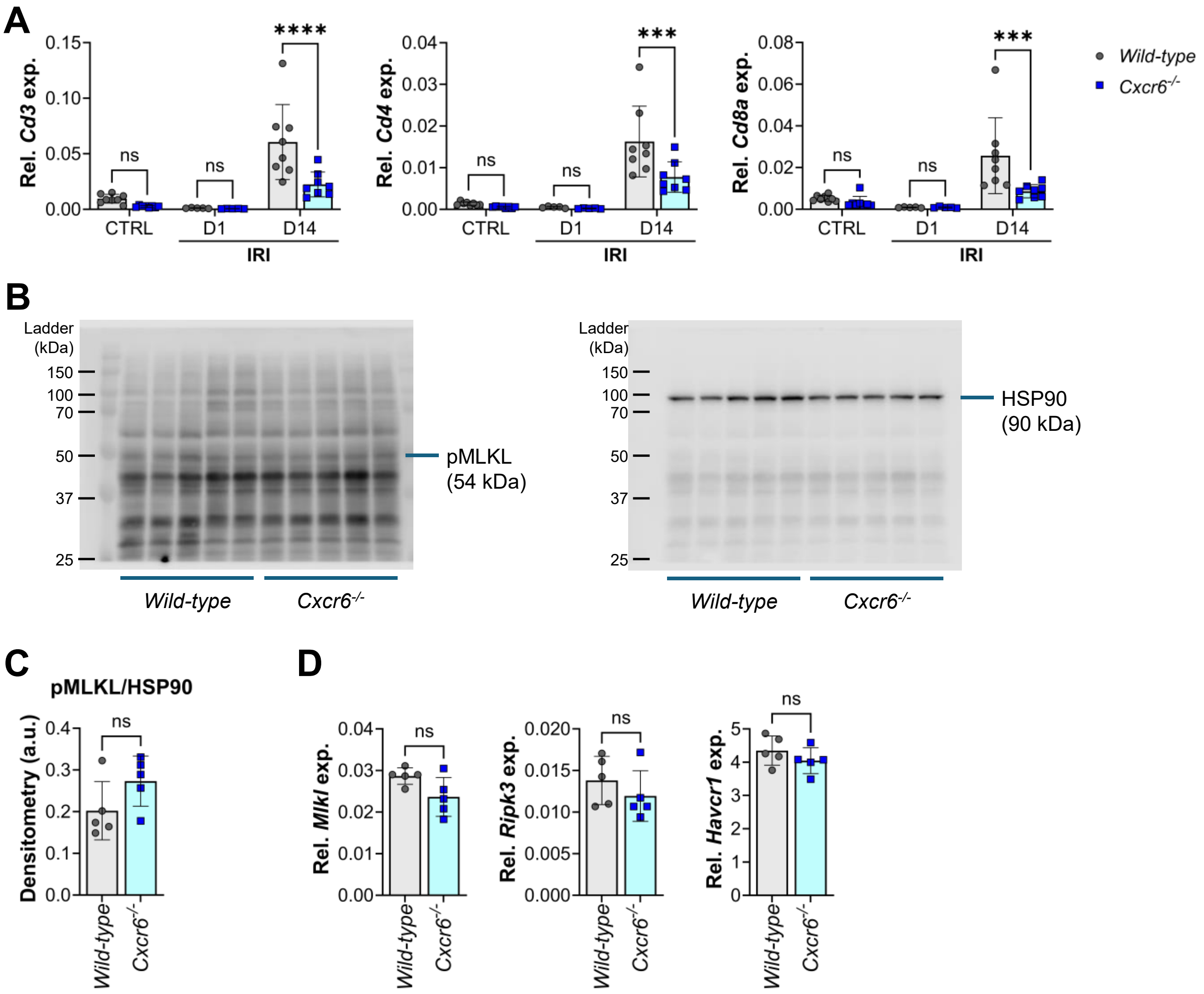

Supplemental Figure 7. CXCR6 deficiency does not attenuate the initial injury following unilateral ischemia/reperfusion injury (U-IRI). (A) *Wild-type* and *Cxcr6*$^{-/-}$ mice were subjected U-IRI. Healthy control (CTRL) or injured (IRI) kidneys were harvested 1 day (n=5 mice/genotype) or 14 days (n=8 mice/genotype) after injury. Quantitative PCR for *Cd3e*, *Cd4*, and *Cd8a* was performed on whole-kidney mRNA. Two-way ANOVA revealed a significant interaction between genotype and time: P=0.0067 (*Cd3e*), P=0.0156 (*Cd4*), and P=0.0168 (*Cd8a*). ***P<0.001 and ****P<0.0001 by Tukey's multiple comparison. ns, not statistically significant. (B) Western blot analysis of phospho-MLKL and HSP90 (loading control, re-probed after stripping) from whole kidney lysates (each lane represents an individual kidney). (C) Densitometric quantification of protein bands. Expression of phospho-MLKL was normalized to HSP90. n=5 kidneys/genotype. ns, not statistically significant by unpaired *t* test. (D) Quantitative PCR for *Mlkl*, *Ripk3*, and *Havcr1* was performed on whole-kidney mRNA. ns, not statistically significant by unpaired *t* test.

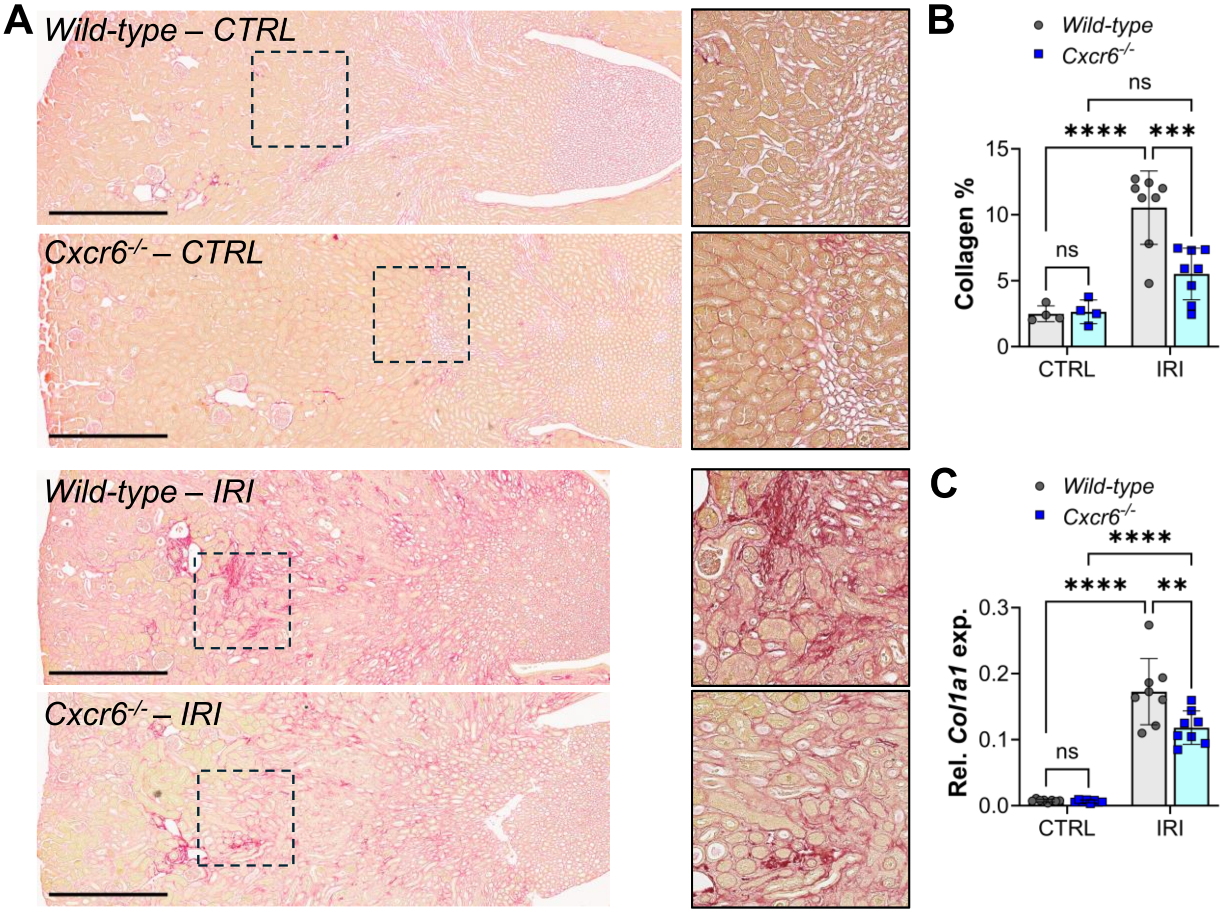

Supplemental Figure 8. CXCR6 promotes interstitial fibrosis in late-stage unilateral ischemia/reperfusion injury (U-IRI). *Wild-type* and *Cxcr6*$^{-/-}$ mice were subjected U-IRI. Healthy control (CTRL) or injured (IRI) kidneys were harvested 14 days after U-IRI. (A-B) Picrosirius red staining was performed on the whole kidney sections from CTRL and IRI kidneys. The stained slides were scanned using Aperio LV1 Real-time slide scanner and processed using ImageScope software. Representing images were shown on the same scale. Scale car: 0.5 mm. (B) The percentage of Picrosirius red-positive area was quantified using ImageJ. P=0.0127 (genotype), P<0.0001 (injury factor), and P=0.0087 (injury and genotype interaction) by two-way ANOVA. **P<0.01 and ****P<0.0001 by Tukey's multiple comparison. ns, not statistically significant. (C) Quantitative PCR for *Col1a1* was performed on whole-kidney mRNA. P=0.0091 (genotype), P<0.0001 (injury factor), and P=0.0123 (injury and genotype interaction) by two-way ANOVA. **P<0.01 and ****P<0.0001 by Tukey's multiple comparison. ns, not statistically significant.

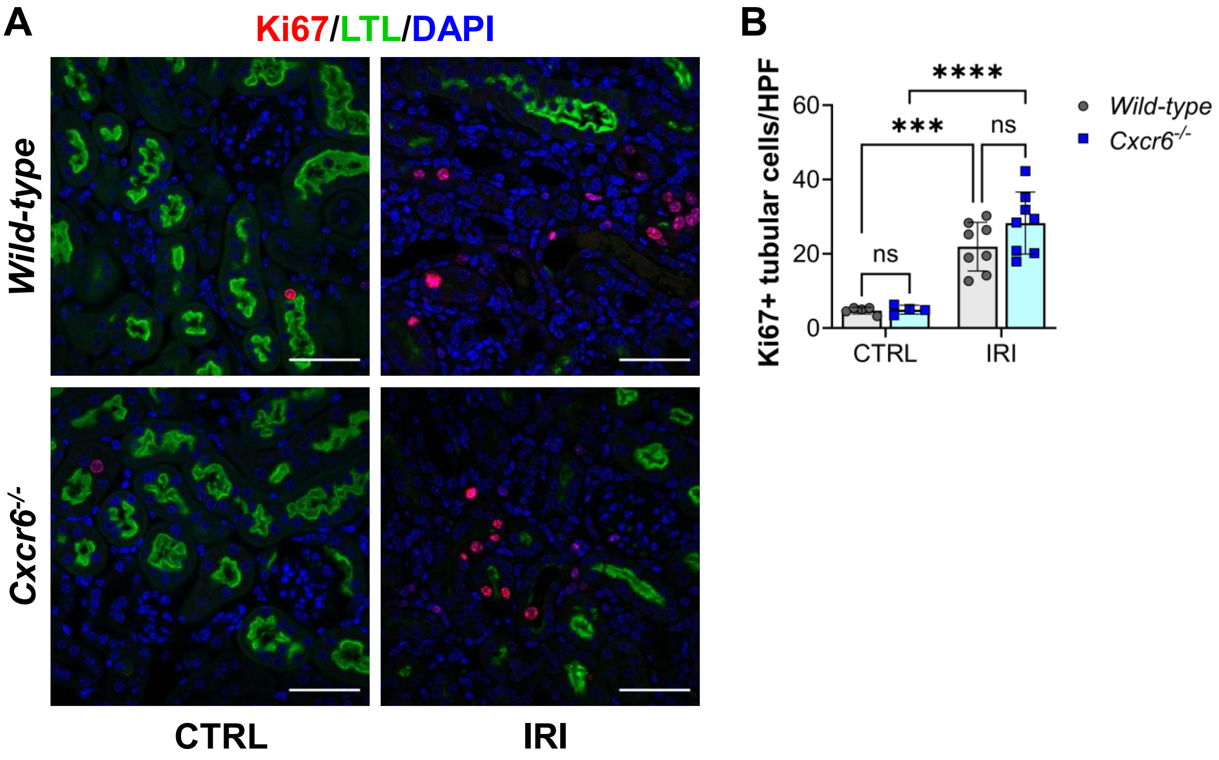

Supplemental Figure 9. CXCR6 deficiency does not alter tubular proliferation at the late stage of ischemia/reperfusion injury (U-IRI). *Wild-type* and *Cxcr6*$^{-/-}$ mice were subjected to unilateral ischemia/reperfusion injury (U-IRI), and the injured kidneys were harvested on day 14 post-injury. Control (CTRL) kidneys were obtained from healthy uninjured mice. (A) Kidney sections were immunofluorescence-stained with Ki67 (red), LTL (green), and DAPI (blue). Original magnification, ×63. Scale bar: 50 μm. (B) Quantitation of Ki67+ tubular cell per high power field (HPF). Two-way ANOVA revealed no significant interaction between genotype and injury ($P=0.2516$). ***$P<0.001$ and ****$P<0.0001$ by Tukey's multiple comparison. ns, not statistically significant.

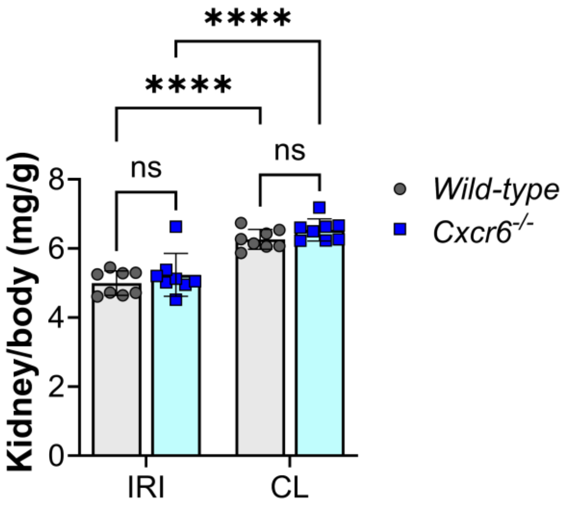

Supplemental Figure 10. Kidney-to-body weight ratio. *Wild-type* and *Cxcr6*$^{-/-}$ mice were subjected U-IRI. The injured (IRI) and contralateral (CL) kidneys were harvested and weighted 14 days after U-IRI. P=0.0934 (genotype), P<0.0001 (injury factor), and P=0.9089 (injury and genotype interaction) by two-way ANOVA. ****P<0.0001 by Tukey's multiple comparison. ns, not statistically significant.

References

1. Xu L, Guo J, Moledina DG, Cantley LG. Immune-mediated tubule atrophy promotes acute kidney injury to chronic kidney disease transition. *Nat Commun* 2022, **13**(1)**:** 4892.
